# Dynamic changes in microbial communities and flavor during different fermentation stages of proso millet Baijiu, a new product from Shanxi light-flavored Baijiu

**DOI:** 10.3389/fmicb.2024.1333466

**Published:** 2024-01-22

**Authors:** Jia Zhao, Zhenfeng Gao

**Affiliations:** ^1^Department of Biological Science and Technology, Jinzhong University, Jinzhong, China; ^2^College of Food Science and Engineering, Shanxi Agriculture University, Jinzhong, China

**Keywords:** proso millet, Baijiu, fermentation, microbial community, flavor

## Abstract

**Introduction:**

Proso millet, a high-quality fermentation material used for Chinese yellow wine production, can produce special flavored substances; however, its role in improving the flavor and altering microbial communities of light-flavored Baijiu during fermentation remain unknown. Thus, we aimed to investigate the effect of proso millet on improving the flavor of light-flavored Baijiu and altering microbial communities during different fermentation stages.

**Methods:**

The dynamic changes in the microbial communities and flavor of proso millet (50%) + sorghum (50%) mixed fermentation samples were analyzed through intermittent sampling on days 7, 14, 21, and 28 of the fermentation process. Microbial high-throughput sequencing and the analysis of flavor characteristics were conducted through 16S DNA/ ITS amplicon sequencing and gas chromatography (multi-capillary column)-ion mobility spectrometry, respectively.

**Results:**

Proso millet significantly changed the core flavor compound composition of traditional light-flavored Baijiu from ethyl acetate, ethyl hexanoate, ethyl hexanoate dimer, ethyl butanoate, ethyl lactate, and butyl acetate to oct-2-ene, 2-butanol, propyl propanoate, 2-pentenal, and 4-methylpentanal. The amplicon sequencing analysis revealed that the alpha diversity parameters of bacterial and fungal communities, including the Chao1, Pielou_e, Shannon, and Simpson indices, for proso millet–sorghum mixed fermentation samples were significantly higher than those for sorghum fermentation samples (*p* < 0.05). Of the 40 most significant microbial genera in two treatments, proso millet significantly increased the abundance of 12 bacterial and 18 fungal genera. Among the 40 most significant bacterial and fungal species, 23 bacterial species belonged to the *Lactobacillus* genus, whereas the 30 primary fungal species belonged to 28 different genera. The analysis of the relationship between microbial changes and the main flavor compounds of light-flavored Baijiu showed that bacteria from the *Weissella*, *Acinetobacter*, *Bacteroides*, *Psychrobacter*, *Pseudarthrobacter*, *Lactococcus*, *Chloroplast*, *Saccharopolyspora*, *Psychrobacter*, *Saccharopolyspora*, *Pseudonocardiaceae*, *Bacteroides* genera and fungi from the *Thermoascus*, *Aspergillus*, *Pichia*, *Rhizomucor*, *Papiliotrema*, *Hyphopichia*, and *Mucor* genera significantly inhibited the synthesis of ethyl hexanoate, ethyl butanoate, ethyl lactate ethyl lactate, and butyl acetate but increased the synthesis of ethyl acetate (*p* < 0.05). Moreover, these microbes exhibited a significantly greater abundance in proso millet–sorghum mixed fermentation samples than in sorghum samples. The synthesis of special flavored compounds in proso millet Baijiu was significantly positively correlated with the presence of fungi from the *Rhizopus*, *Papiliotrema*, *Wickerhamomyces*, *Aspergillus*, and *Thermoascus* genera but negative correlated with the presence of bacteria from the *Weissella*, *Acinetobacter*, *Psychrobacter*, *Pseudarthrobacter*, *Bacteroides*, and *Saccharopolyspora* genera. Regarding ethanol content, the low alcohol content of Fenjiu may be due to the significantly high abundance of fungi from the *Psathyrella* genus and bacteria from the *Staphylococcus*, *Kroppenstedtia*, *Brevibacterium*, and *Acetobacter* genera during fermentation. In summary, proso millet significantly altered the flavor of light-flavored Baijiu by inducing the formation of a special microbial community; however, it did not increase alcohol concentration.

**Discussion:**

This study lays the foundation for future research on Baijiu fermentation. Additionally, the study findings may help improve the production efficiency and elevate the quality and flavor of the final product.

## 1 Introduction

In China, Baijiu has evolved from being a mere beverage to a symbol of culture. The Baijiu culture in China has a rich historical background, and companies that produce distilled Baijiu operate on a large scale with huge profits. Fenjiu, traditional Chinese Baijiu primarily made from non-glutinous sorghum, is highly popular light aroma-style beverage that has been consumed for over 1,500 years (Ma et al., 2013). However, companies that produce light-flavored Baijiu face several challenges, including low yield, limited high-end product options, declining quality, and a stagnant market due to a narrow flavor profile, which have hindered their development (Yifan et al., 2021). Currently, researchers are actively exploring methods to enhance their understanding of light-flavored Baijiu and improve its quality, with research focusing on aspects such as the microbial community structure in light-flavored Daqu, flavor chemistry of light-flavored Baijiu, influence of sorghum varieties on the flavor of light-flavored Baijiu, and the relationship between light-flavored Baijiu and microorganisms (Liu and Sun, 2018; Huang et al., 2020; Zhang et al., 2022; Xiang et al., 2023; Zhang et al., 2023). In addition, studies have examined the physicochemical properties of Chinese light-flavored Baijiu during storage and explored ways to improve sorghum varieties for Baijiu production (Ma et al., 2013; Kang et al., 2020). Furthermore, to improve the quality of light-flavored Baijiu, some other traditional brewing materials such as rice, barley, and glutinous sorghum, have been currently added to the raw materials used for its production. However, it remains unclear whether proso millet, which is the primary raw material for Chinese yellow wine production, can improve the flavor of light-flavored Baijiu. In addition, the microbial communities and flavor profiles of sorghum-based light-flavored Baijiu during fermentation have not yet been determined.

Proso millet (*Panicum miliaceum* L.) is an annual herbaceous cereal crop, commonly known as “huangmi” in North and Northwest China; it has a high nutritional value and is rich in starch, dietary fibers, proteins, phenolic compounds, vitamins, amino acids, and trace elements (Kalam Azad et al., 2019; Wiedemair et al., 2020; Yuan et al., 2022). It is extensively cultivated in arid or semi-arid regions across North China, Asia, Australia, North America, Europe, and Africa (Sanderson et al., 2017; Yang et al., 2018; Kučera et al., 2019; Gong et al., 2021; Narciso and Nyström, 2023). Moreover, proso millet has demonstrated antioxidant, anti-proliferative, antimicrobial, and anti-carcinogenic properties. It has also been associated with alleviating celiac disease, regulating blood glucose and cholesterol levels, and preventing diabetes and cardiovascular disorders (Kim et al., 2011; Sanderson et al., 2017; Shen et al., 2018; Yang et al., 2018; Kalam Azad et al., 2019; Agarwal and Chauhan, 2019). Consequently, proso millet is a valuable resource for the development of healthy foods, and has recently been used to produce popular products, such as flour, bread, couscous, biscuits, porridge, extruded snacks, and fermented beverages (Sanderson et al., 2017; McSweeney et al., 2017a; Wang J. et al., 2020). To promote the development of new products, increase awareness regarding the functional properties of proso millet, and determine the potential applications of proso millet in pasta processing or other cooking processes, significant research attention has been devoted to topics such as drought resistance, cultivation methods, yield improvement, breeding, functional properties, physicochemical characteristics, nutritional composition, protein properties, starch physicochemical properties, and digestibility of the crop, as well as its biological structure and microstructure analyses (Gulati et al., 2017, 2018; Hasseldine et al., 2017; McSweeney et al., 2017b; Balkrishna and Visvanathan, 2019; Johnson et al., 2019; Yang et al., 2019; Liu et al., 2020; Wiedemair et al., 2020; Gong et al., 2021; Chang et al., 2023; Kumar et al., 2023). However, limited research has been carried out on its fermentation characteristics and its use in the development of fermented products. Despite the development of healthy proso millet wines (yellow wine or fermented alcoholic drinks), beer, and vinegar, with recent studies shedding light on their flavor composition and fermentation processes (Liu et al., 2018; Santra et al., 2019; Zheng et al., 2020; Bian et al., 2022), there is limited research on new fermentation products, particularly hard liquor made from proso millet. Moreover, studies on the relevant fermentation processes, flavor profiles, and mechanisms underlying quality formation for proso millet liquor are limited.

Therefore, in recent years, our research team has optimized the fermentation formula and technology based on Fenjiu Daqu, leading to the successful development of a new product (ZL201410293583.1). In this study, we aimed to investigate the effects of proso millet on improving the flavor of light-flavored Baijiu by studying the microbial composition and flavor differences between the new product and sorghum-based light-flavored Baijiu during fermentation. In addition, we aimed to reveal the mechanistic correlations between microbial community composition and flavor formation. Our findings may help expand the application range of proso millet and provide a reference basis for improving the flavor of Fenjiu or other light-flavored Baijiu beverages.

## 2 Materials and methods

### 2.1 Experimental design and sampling

Sorghum (Jinza No. 22) and proso millet (Jinshu No. 9) were procured from Shanxi Jinliang Agricultural Development Company, Xiyang County, Jinzhong City, Shanxi Province, China. Two fermentation tests, sorghum and mixed material (composition of 50% sorghum and 50% proso millet) fermentation, were conducted under commercial conditions at the Xinghua Village Fen Distillery in Shanxi Province. Samples of fermented grains (FG) were collected at four different time points during alcoholic fermentation in the pit: days 7, 14, 21, and 28. To ensure comprehensive data and representativeness, each sample at every time point comprised nine subsamples collected from various spatial positions within the underground vats, with each subsample weighing 100 g (Supplementary Figure S1). Three parallel samples (from three random cellars) were collected for each sample type. Subsequently, all 24 samples were transported to the laboratory on ice and stored at −80°C until further analysis. The mixed proso millet fermentation samples collected on days 7, 14, 21, and 28 of fermentation were designated as MA, MB, MC, and MD, respectively. The sorghum fermentation samples collected on days 7, 14, 21, and 28 of fermentation were designated as GA, GB, GC, and GD, respectively.

### 2.2 Microbiological analysis

#### 2.2.1 DNA extraction and high-throughput sequencing analysis

Total genomic DNA was extracted from 200 mg of FG samples using Fast DNA SPIN extraction kits (MP Biomedicals, Santa Ana, CA, USA), following the manufacturer’s protocol. The quantity and quality of the extracted DNA were assessed using an ND-1000 spectrophotometer (Thermo Fisher Scientific, Waltham, MA, USA) and agarose gel electrophoresis, respectively.

For bacterial analysis, the V3-V4 hypervariable region of 16S rRNA genes was amplified using the universal primer pair, 338F (5′-ACTCCTACGGGAGGCAGCA-3′) and 806R (5′-GGACTACHVGGGTWTCTAAT-3′), each with unique barcodes (Lin et al., 2018). Sample-specific 7-bp barcodes were incorporated into the primers for multiplex sequencing. Polymerase chain reactions (PCRs) were conducted in a 25-μL reaction volume, consisting of 5 μL of Q5 reaction buffer (5×), 5 μL of Q5 high-fidelity GC buffer (5×), 0.25 μL of Q5 high-fidelity DNA polymerase (5 U/μL), 2 μL (2.5 mM) of deoxynucleotide triphosphates, 1 μL (10 μM) of each forward and reverse primer, 2 μL of DNA template, and 8.75 μL of double distilled water. The thermal cycle profile included an initial 30 s of denaturation at 98°C, followed by denaturation at 98°C for 15 s, annealing at 50°C for 30 s, and extension at 72°C for 30 s, with final extension at 72°C for 5 min.

For fungal analysis, the ITS1 region was targeted using the primer ITS5 (5′-GGAAGTAAAAGTCGTAACAAGG-3′), and the reverse primer, ITS2 (5′-GCTGCGTTCTTCATCGATGC-3′), following a previously published method (Huang et al., 2016). The PCR volume and conditions were the same as those for the bacterial analysis.

PCR amplicons were purified using Agencourt AMPure Beads (Beckman Coulter, Indianapolis, IN, USA) and quantified using the PicoGreen dsDNA Assay Kit (Invitrogen, Carlsbad, CA, USA). Following individual quantification, amplicons were pooled in equal amounts, and pair-end 2 × 300 bp sequencing was conducted on the Illumina MiSeq platform using MiSeq Reagent Kit v3, carried out by Shanghai Personal Biotechnology Co., Ltd. (Shanghai, China).

#### 2.2.2 Bioinformatics and statistical analysis

The Quantitative Insights Into Microbial Ecology (QIIME, v1.8.0) pipeline was employed to process sequencing data, following established procedures (Caporaso et al., 2010). In summary, raw sequencing reads that precisely matched the barcodes were assigned to their respective samples and identified as valid sequences. Paired-end DNA fragments were initially assembled using FLASH (Zhang et al., 2014). After chimera detection, the remaining high-quality sequences were clustered into operational taxonomic units (OTUs) with 97% sequence identity using UCLUST (Jiang et al., 2012). Taxonomic assignments for representative sequences from each OTU were determined using the Silva database (16S rDNA) and Unite database (ITS) within the QIIME platform (Caporaso et al., 2010). Data analysis was primarily conducted using the QIIME and R packages (v3.2.0). Alpha diversity indices at the OTU level, including the Chao1 richness estimator, the abundance-based coverage estimator metric, the Shannon diversity index, and the Simpson’s index, were calculated using the OTU table in QIIME. Differences in microbiota structure among groups were assessed using permutational multivariate analysis of variance and analysis of similarities using the “vegan” function in R (Takeshita et al., 2012; Kelly et al., 2015). Taxonomic compositions and abundance were visualized using MEGAN and GraPhlAn (Li et al., 2019). Taxa abundances at the phylum, class, order, family, genus, and species levels were statistically compared among samples or groups using Metastats (Elokil et al., 2020) and visualized using violin plots. Raw sequence data have been deposited in the NCBI Sequence Read Archive under the accession numbers, PRJNA1015004, PRJNA1014983, PRJNA1013705, and PRJNA1014132.

### 2.3 Flavor analysis

The FG samples used for flavor analysis were the same as those mentioned in Section 2.1. Volatile compounds were detected using FlavourSpec® (G.A.S., Germany) with an FS-SE-54-CB-1 column (15 m length × 0.53 mm inner diameter) via GC-IMS. Briefly, 2 g of each fermented sample was placed in a 20-mL headspace bottle, incubated at 60°C, and centrifuged at 500 rpm for 15 min. Subsequently, 200 μL of liquid was extracted from the headspace bottle using an 85°C syringe and automatically injected into the injector of the GC-IMS equipment. Nitrogen gas with 99.999% purity was used as the carrier gas, and volatile organic compounds (VOCs) in the FG samples were separated using a 60°C quartz capillary column. The GC flow conditions were as follows: 2 mL/min for 2 min, 100 mL/min for 20 min, 150 mL/min for 30 min, and then the flow was halted. VOC separation was carried out at a column temperature of 60°C, with detection at 45°C in the IMS ionization chamber at a drift gas flow rate of 150 mL/min. All experiments were conducted in triplicate.

### 2.4 Statistical analyses

Statistical analyses were performed using one-way analysis of variance in the SPSS version 17.0 software (SPSS, Inc., Chicago, IL, USA). Differences between groups were evaluated using Duncan’s multiple comparison tests, and *p* < 0.05 was considered statistically significant.

## 3 Results

### 3.1 Microbiological analysis

#### 3.1.1 Overall analysis of Illumina MiSeq data

In total, 324,421 and 292,751 high-quality sequences were obtained from the bacterial 16S rRNA V3-V4 sequences, whereas 514,202 and 470,916 high-quality sequences were obtained from the fungal ITS sequences for sorghum and proso millet FG samples, respectively. These sequences were clustered into 3,133 and 371 (sorghum FG samples) and 7,255 and 574 (proso millet FG samples) amplicon sequence variants (ASVs), respectively, at a 97% similarity level. The coverage estimator for bacterial and fungal ASVs exceeded 99% for all samples, indicating that the obtained sequence reads adequately represented the microbial diversity.

Microbial community richness, diversity, and evenness in sorghum and proso millet FG samples were assessed using the Chao1, Shannon, Simpson, and Pielou’s evenness indices (Table 1). Microbial diversity in both sorghum and proso millet FG samples decreased as fermentation time increased. Despite showing a lower number of high-quality sequences, proso millet FG samples exhibited a higher microbial diversity than sorghum FG samples as the bacterial Chao1, Shannon, Pielou’s evenness, and Simpson indices for proso millet FG samples at different stages of fermentation were approximately twice as those for sorghum FG samples; in addition, the fungal Chao1 index was considerably higher for proso millet FG samples than for sorghum FG samples (Table 1).

**Table 1:** Summary of diversity indices for microbial communities based on 16S rRNA and ITS1 gene sequencing data.

Gene	Sample ID	Chao1	Pielou_e	Shannon	Simpson
16S rRNA	GA	1186.40 ± 262.55b	0.60 ± 0.01b	6.00 ± 0.33b	0.91 ± 0.01b
	GB	455.02 ± 60.10c	0.27 ± 0.00d	2.35 ± 0.08d	0.42 ± 0.01d
	GC	457.68 ± 24.80c	0.25 ± 0.00de	2.12 ± 0.04d	0.37 ± 0.01e
	GD	458.70 ± 68.53c	0.23 ± 0.02e	1.98 ± 0.20d	0.35 ± 0.04e
	MA	1834.42 ± 213.56a	0.66 ± 0.03a	7.08 ± 0.49a	0.96 ± 0.01a
	MB	1698.85 ± 95.77a	0.65 ± 0.01a	6.88 ± 0.12a	0.96 ± 0.00a
	MC	1623.78 ± 114.02a	0.59 ± 0.02b	6.17 ± 0.29b	0.89 ± 0.02b
	MD	1094.76 ± 6.62b	0.44 ± 0.01c	4.29 ± 0.12c	0.69 ± 0.01c
ITS	GA	58.65 ± 7.69d	0.33 ± 0.04ab	1.95 ± 0.32 cd	0.64 ± 0.08c
	GB	62.53 ± 2.79c	0.25 ± 0.00c	1.49 ± 0.04e	0.53 ± 0.01d
	GC	80.84 ± 15.43b	0.32 ± 0.04ab	2.00 ± 0.18bcd	0.64 ± 0.04c
	GD	85.15 ± 1.87ab	0.29 ± 0.02bc	1.86 ± 0.10d	0.59 ± 0.04c
	MA	100.59 ± 8.52ab	0.29 ± 0.01bc	1.94 ± 0.02 cd	0.66 ± 0.01c
	MB	104.97 ± 11.39a	0.35 ± 0.01a	2.34 ± 0.05a	0.72 ± 0.01a
	MC	98.90 ± 14.31ab	0.35 ± 0.03a	2.30 ± 0.24ab	0.72 ± 0.01a
	MD	95.78 ± 20.14ab	0.34 ± 0.02ab	2.20 ± 0.18abc	0.70 ± 0.03ab

All data are presented as mean ± standard deviation (*n* = 3). Values with different letters in a column indicate significant differences at *p* < 0.05 as determined by one-way ANOVA Duncan’s test.

#### 3.1.2 Bacterial community composition during proso millet Baijiu fermentation

**Figure 1:**
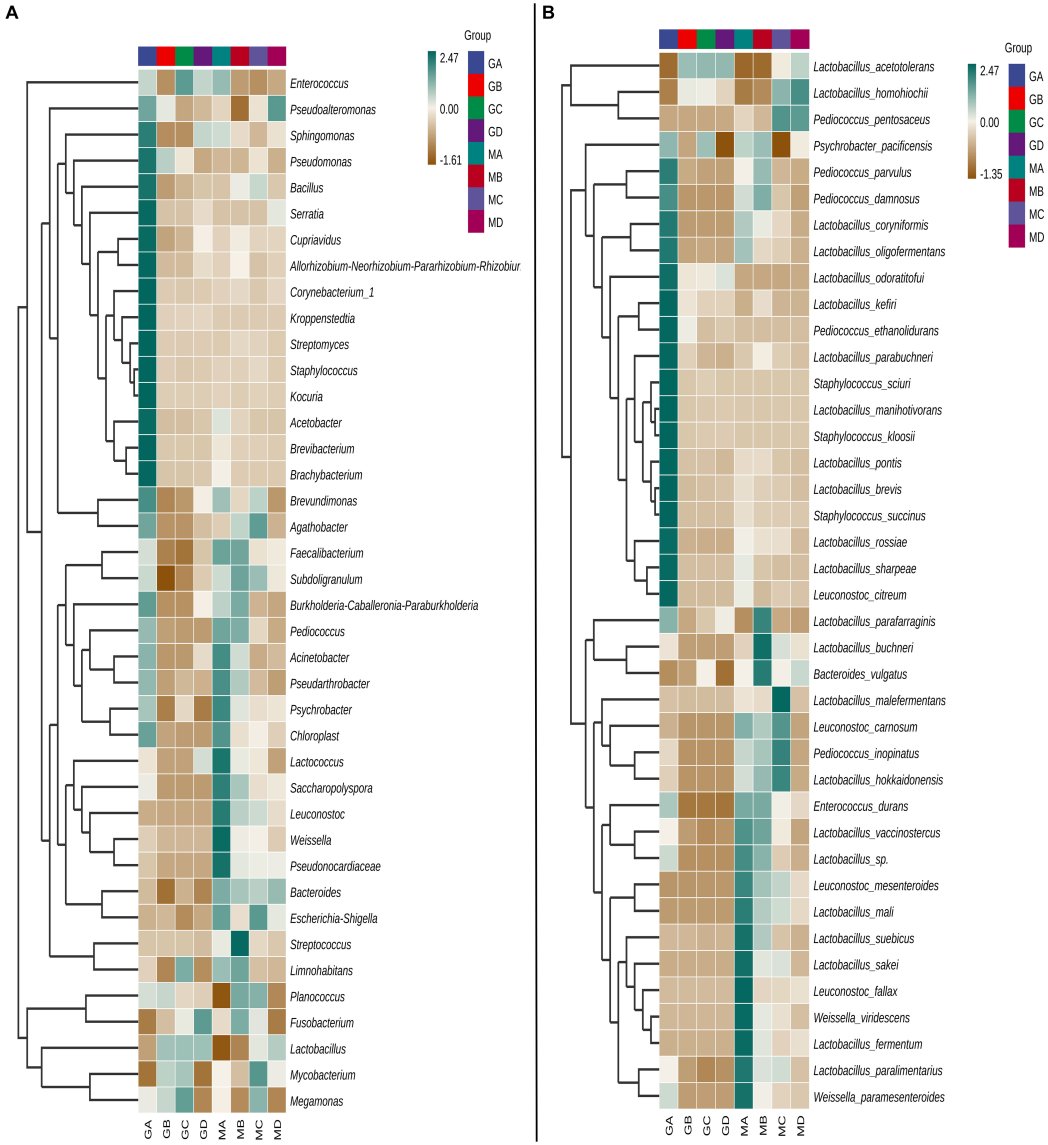
Top 40 bacterial genera **(A)** and species **(B)** obtained from the samples MA–MD and GA–GD.

At the genus level, the bacterial community composition in samples MA–MD differed significantly from that in samples GA–GD (Figure 1A). Compared to those in sorghum fermentation samples (GA–GD), the core bacteria genera in proso millet fermentation samples changed from *Lactobacillus*, *Megamonas*, and *Enterococcus* to *Bacteroides*, *Escherichia-Shigella*, *Leuconostoc*, *Subdoligranulum*, and *Faecalibacterium*. However, *Lactobacillus* species played a major role in both proso millet and sorghum Baijiu fermentation. For MA samples, the abundance of bacteria from the genera *Acinetobacter*, *Pseudarthrobacter*, *Psychrobacter*, *Chloroplast*, *Lactococcus*, *Saccharopolyspora*, *Leuconostoc*, *Weissella*, and *Pseudonocardiaceae* was considerably higher than that of bacteria from other genera. However, the abundance of bacteria belonging to these nine genera gradually decreased in MB–MD samples. The *Acinetobacter*, *Pseudarthrobacter*, *Psychrobacter*, and *Chloroplast* genera were predominant in GA samples; however, their abundance was lower than that of other genera, such as the *Sphingomonas*, *Pseudomonas*, *Bacillus*, *Serratia*, *Streptomyces*, *Staphylococcus*, *Cupriavidus*, *Allorhizobium-Neorhizobium-Pararhizobium-Rhizobium*, *Corynebacterium*, *Kocuria*, *Kroppenstedtia*, *Acetobacter*, *Brevibacterium*, and *Brachybacterium*. In MB samples, bacteria from the *Streptococcus*, *Limnohabitans*, *Planococcus*, *Fusobacterium*, *Faecalibacterium*, *Subdoligranulum*, *Burkholderia-Caballeronia-Paraburkholderia*, and *Pediococcus* genera dominated, with their abundance surpassing that in GB samples. Genera such as *Agathobacter*, *Subdoligranulum*, *Escherichia-Shigella*, *Planococcus*, *Mycobacterium*, and *Megamonas* were prevalent in MC samples. MC samples were mainly characterized by *Megamonas*, *Limnohabitans*, *Lactobacillus*, *Mycobacterium*, and *Enterococcus* species. In contrast, MD samples exhibited a dominance of the genera, *Pseudoalteromonas*, *Bacteroides*, and *Lactobacillus*. GD samples were characterized by the genera, *Fusobacterium*, and *Lactobacillus*.

At the species level, the core bacterial composition in mixed fermentation samples was significantly different from that in sorghum fermentation samples. Species such as *Leuconostoc carnosum*, *Pediococcus inopinatus*, *Lactobacillus hokkaidonensis*, and *Leuconostoc mesenteroides* were the core bacteria identified in mixed fermentation samples, whereas species such as *Lactobacillus acetotolerans* and *Lactobacillus homohiochii* were the core bacteria identified in sorghum fermentation samples at different fermentation stages (Figure 1B).

Of the 40 most significant species, 23 belonged to the *Lactobacillus* genus. However, species composition differed between proso millet and sorghum fermentation samples (Figure 1B). In MA samples, species such as *L. carnosum*, *Enterococcus durans*, *Lactobacillus vaccinostercus*, *L. mesenteroides*, *Lactobacillus mali*, *Lactobacillus suebicus*, *Lactobacillus sakei*, *Leuconostoc fallax, Weissella viridescens*, *Lactobacillus fermentum*, *Lactobacillus paralimentarius*, and *Weissella paramesenteroides* were predominant. In contrast, GA samples were characterized by species such as *Pediococcus parvulus*, *Pediococcus damnosus*, *Lactobacillus coryniformis*, *Lactobacillus oligofermentans*, *Lactobacillus odoratitofui*, *Lactobacillus kefiri*, *Pediococcus ethanolidurans*, *Lactobacillus parabuchneri*, *Staphylococcus sciuri*, *Lactobacillus manihotivorans*, *Staphylococcus kloosii*, *Lactobacillus pontis*, *Lactobacillus brevis*, *Staphylococcus succinus*, *Lactobacillus rossiae*, *Lactobacillus sharpeae*, and *Leuconostoc citreum*, which was significantly different from the composition of MA samples. In MA samples, in total, six, three, two, and one species belonged to *Lactobacillus*, *Leuconostoc*, *Weissella*, and *Enterococcus* genera, respectively, were identified. In MB samples, the abundance of species such as *Lactobacillus parafarraginis*, *Lactobacillus buchneri*, and *Bacteroides vulgatus* was significantly higher than that of other species and that in GB samples. In contrast, species such as *Lactobacillus malefermentans*, *L. carnosum*, *P. inopinatus*, *L. hokkaidonensis*, *L. homohiochii*, and *Pediococcus pentosaceus* were the primary species in MC samples. In MD samples, *L. homohiochii* and *P. pentosaceus* dominated. *L. acetotolerans* was the core species in samples GB, GC, and GD (Figure 1B).

#### 3.1.3 Fungal community composition during proso millet Baijiu fermentation

**Figure 2:**
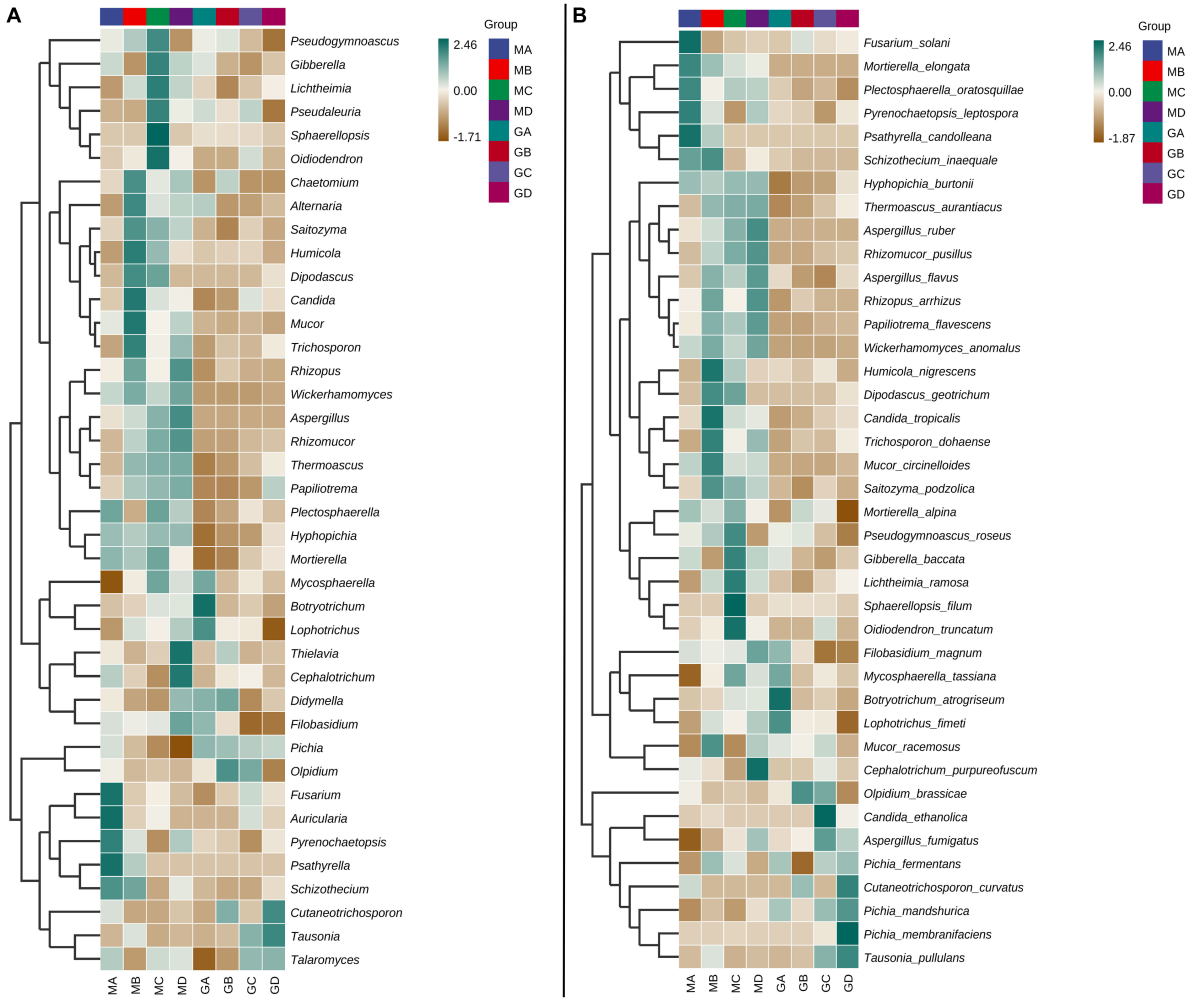
Top 40 fungal genera **(A)** and species **(B)** obtained from the samples MA–MD and GA–GD.

At the genus level, the fungal community composition of MA–MD samples exhibited significant differences from that of GA–GD samples (Figure 2A). Compared to those in sorghum fermentation samples (GA–GD), core fungal genera in proso millet fermentation samples changed from *Pichia*, *Olpidium*, *Tausonia*, *Talaromyces*, *Cutaneotrichosporon*, and *Didymella* to *Wickerhamomyces*, *Rhizopus*, *Aspergillus*, *Rhizomucor*, *Hyphopichia*, *Mortierella*, *Papiliotrema*, *Plectosphaerella*, *Mucor*, *Trichosporon*, *Candida*, *Chaetomium*, *Gibberella*, and *Pseudogymnoascus*. In addition, the main genera identified in MA samples were *Fusarium*, *Auricularia*, *Pyrenochaetopsis*, *Psathyrella*, and *Schizothecium*; however GA samples were characterized by dominant genera such as *Mycosphaerella*, *Botryotrichum*, and *Lophotrichus*. MB samples included *Chaetomium*, *Alternaria*, *Saitozyma*, *Humicola*, *Dipodascus*, *Candida*, *Mucor*, and *Trichosporon* as the main genera. However, GB samples contained fungi from the *Olpidium*, *Didymella*, and *Cutaneotrichosporon* genera. In contrast, MC samples exhibited a different profile, with genera such as *Pseudogymnoascus*, *Gibberella*, *Lichtheimia*, *Pseudaleuria*, *Sphaerellopsis*, and *Oidiodendron* being predominant. At the time of GC sample collection, the dominant genera had shifted to *Olpidium*, *Tausonia*, and *Talaromyces*. Finally, MD samples exhibited *Rhizopus*, *Wickerhamomyces*, *Aspergillus*, *Rhizomucor*, *Thielavia*, *Cephalotrichum*, and *Filobasidium* as main genera. The *Cutaneotrichosporon* and *Tausonia* genera were the dominant genera in GD samples (Figure 2A).

At the species level, specific species were associated with different stages of fermentation. For proso millet + sorghum mixed fermentation samples, there were 23 typical core species: *Hyphopichia burtonii*, *Thermoascus aurantiacus*, *Plectosphaerella oratosquillae*, *Pyrenochaetopsis leptospora*, *Mortierella elongate*, *Aspergillus ruber*, *Rhizomucor pusillus*, *Aspergillus flavus*, *Rhizopus arrhizus*, *Papiliotrema flavescens*, and *Wickerhamomyces anomalus*. However, only *Pichia fermentans*, *Pichia mandshurica*, *Pichia membranifaciens*, *Tausonia pullulans*, and *Olpidium brassicae* were identified as core genera in sorghum fermentation samples based on the analysis of relative abundance (Figure 2B). *Fusarium solani*, *Mortierella elongata*, *P. oratosquillae*, *P. leptospora*, *Psathyrella candolleana*, and *Schizothecium inaequale* were prominent species in MA samples. *T. aurantiacus*, *A. flavus*, *R. arrhizus*, *P. flavescens*, *W. anomalus*, *Humicola nigrescens*, *Dipodascus geotrichum*, *Candida tropicalis*, *Trichosporon dohaense*, *Mucor circinelloides*, *Saitozyma podzolica*, and *Mucor racemosus* were the main species in MB samples, with *S. inaequale* and *M. elongata* present in both MA and MB samples. MC samples exhibited a different set of dominant species, including *S. podzolica*, *Mortierella alpine*, *Pseudogymnoascus roseus*, *Gibberella baccata*, *Lichtheimia ramosa*, *Sphaerellopsis filum*, and *Oidiodendron truncatum*. Furthermore, MD samples featured *T. aurantiacus*, *A. ruber*, *R. pusillus*, *A. flavus*, *R. arrhizus*, *P. flavescens, W. anomalus*, *Filobasidium magnum*, and *Cephalotrichum purpureofuscum*. Across samples MA–MD, 30 dominant species belonging to 28 genera were identified. In contrast, GA samples featured different dominant species, including *F. magnum*, *Mycosphaerella tassiana*, *Botryotrichum atrogriseum*, *Lophotrichus fimeti*, *P. fermentans*, and *P. mandshurica*. GB samples were characterized by *O. brassicae* and *Cutaneotrichosporon curvatus* as main species, whereas GC samples exhibited a different profile, with species such as *O. brassicae*, *Candida ethanolica*, *Aspergillus fumigatus*, *P. mandshurica*, and *T. pullulans* being identified. GD samples exhibited dominant species such as *P. fermentans*, *C. curvatus*, *P. mandshurica*, *P. membranifaciens*, and *T. pullulans* (Figure 2B). Significant differences in high-abundance species were identified between sorghum and millet fermentation samples, with the number of high-abundance species in millet fermentation samples (MA–MD) exceeding that in sorghum fermentation samples (GA–GD) (Figure 2B).

### 3.2 Flavor compound analysis

#### 3.2.1 Flavor compound composition of proso millet Baijiu fermentation samples

**Figure 3:**
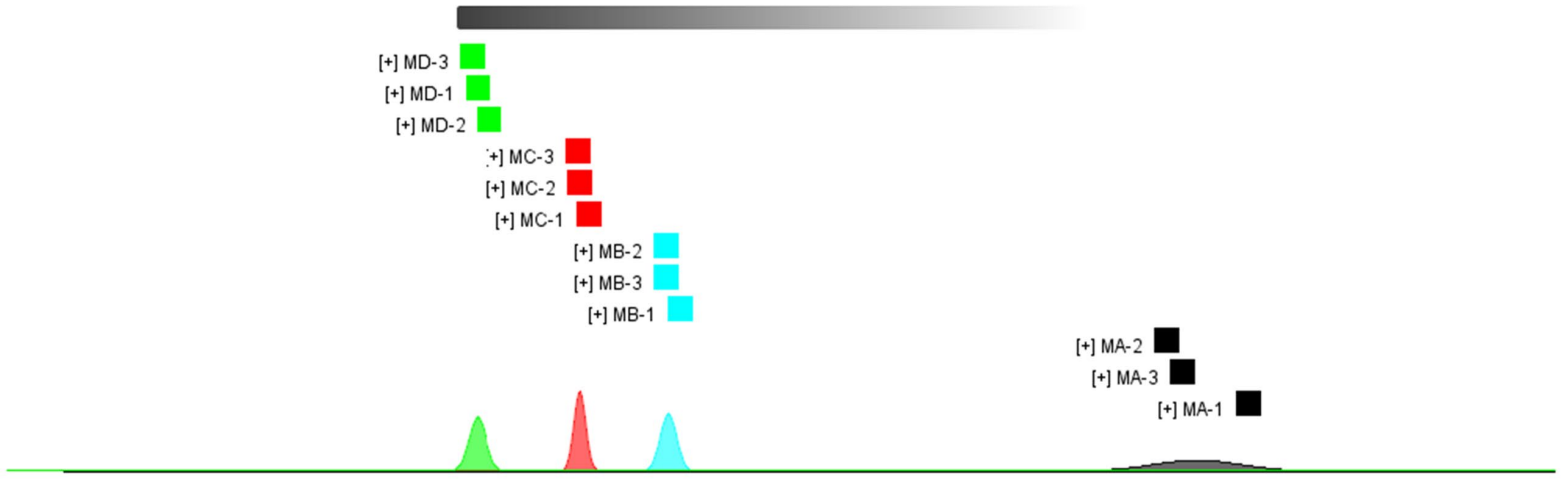
Similarity between MA, MB, MC, and MD samples.

**Figure 4:**
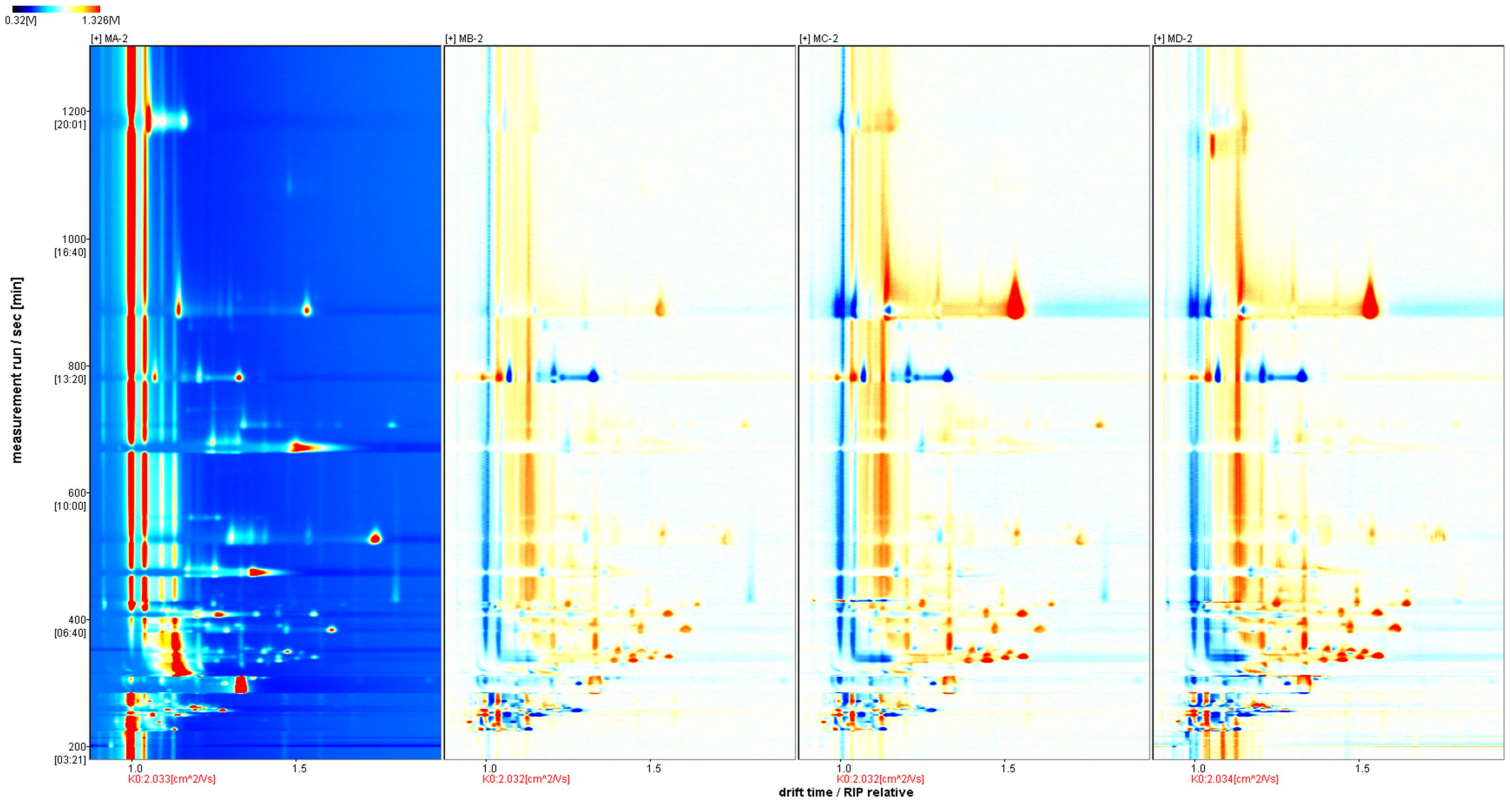
Difference diagram showing the gas phase ion mobility spectrum of the MA, MB, MC, and MD samples. The MA sample was selected as the reference. The spectra of the other samples were obtained from a previous study. If two volatile organic compounds are the same, the deducted background is white, red indicates that the concentration of the substance is higher than that of the reference, and blue indicates that the concentration of the substance is lower than that of the reference.

As shown in Figure 3, there were significant differences among the four samples (MA–MD). The dominant compositions of flavor compounds and their abundances in the four samples were also different. Moreover, compared with MA samples, MD samples exhibited evident differences in the abundances of compounds (Figure 4).

**Figure 5:**
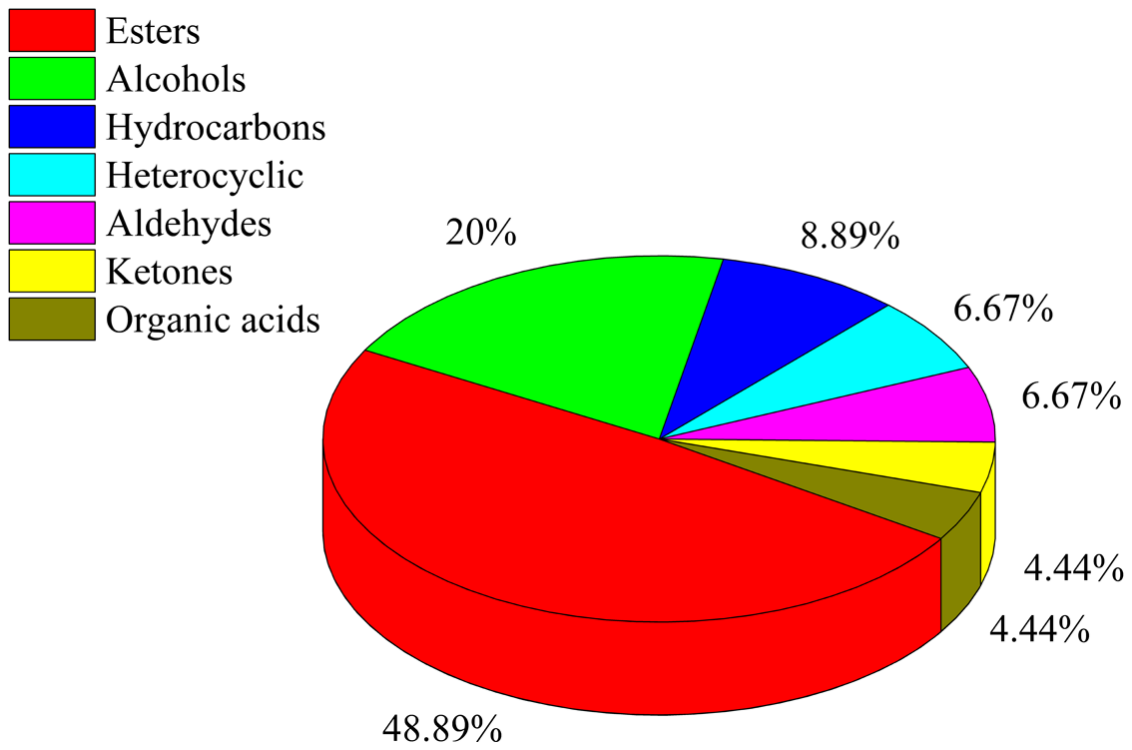
Percentage of seven types of flavor compounds in 46 compounds.

In total, 75 flavor compounds were detected through GC-IMS; however, only 45 of them could be identified based on parameters such as molecular weight, retention index (Ri), retention time (Rt), and mobility time (Dt) (Table 2). Twenty compounds remained unidentified due to similarities in Ri, Rt, and Dt (Supplementary Table S1). Among these 45 compounds, esters were the dominant flavor compounds, constituting 48.89% (22 compounds). Alcohols (nine compounds), hydrocarbons (four compounds), heterocyclic compounds (three compounds), aldehydes (three compounds), ketones (two compounds), and organic acids (two compounds) accounted for 20.00, 8.89, 6.67, 6.67, 4.44, and 4.44% of the compounds, respectively (Figure 5; Table 2).

**Table 2:** Identification of flavor compounds in fermented grain samples at different stages of fermentation based on gas chromatography–ion mobility spectrometry.

Number	Compound	Formula	Molecular weight (MW)	Retention index (Ri)	Retention time (Rt/s)	Mobility time (Dt)	Peak area							
							GA	GB	GC	GD	MA	MB	MC	MD
A1	1-methylethyl acetate	C_5_H_10_O_2_	102.1	870.3	272.4	1.05694	131.6 ± 9.9 b	89.9 ± 8.1 c	86.2 ± 8.0 cd	65.4 ± 8.9 d	293.2 ± 24.7 a	123.9 ± 2.5 b	74.3 ± 1.6 cd	75.1 ± 12.1 cd
A2	tert-butyl acetate	C_6_H_12_O_2_	116.2	732.3	300.5	1.28766	161.0 ± 4.9 b	112.5 ± 5.0 c	113.7 ± 8.1 c	110.1 ± 7.2 c	581.5 ± 38.5 a	172.5 ± 1.6 b	158.3 ± 4.6 b	129.4 ± 2.4 c
A3	Propyl formate	C_4_H_8_O_2_	88.1	642.4	310.0	1.3704	898.9 ± 19.0 a	635.7 ± 21.2 d	718.4 ± 23.7 c	732.1 ± 17.0 bc	741.8 ± 19.4 bc	563.6 ± 9.0 e	764.8 ± 17.0 b	743.7 ± 9.9 bc
A4	Propyl propanoate	C_6_H_12_O_2_	116.2	830.2	394.2	1.22838	208.4 ± 18.6 e	356.1 ± 4.6 d	466.3 ± 23.8 c	444.3 ± 44.2 d	594.1 ± 13.1 b	656.4 ± 2.7 a	583.4 ± 8.5 b	687.3 ± 17.3 a
A5	2-Methylpropyl propionate	C_7_H_14_O_2_	130.2	1070.6	439.7	1.27413	57.8 ± 9.9 b	23.5 ± 2.9 d	20.7 ± 2.1 d	25.2 ± 7.1 cd	24.9 ± 2.2 cd	31.9 ± 5.2 cd	39.4 ± 1.5 c	105.5 ± 17.1 a
A6	Butyl acetate	C_6_H_12_O_2_	116.2	1,070	439.1	1.23993	236.5 ± 56.0 a	29.0 ± 4.0 c	34.3 ± 1.9 bc	34.3 ± 5.4 bc	40.9 ± 2.3 bc	43.5 ± 3.4 bc	41.0 ± 4.9 bc	70.6 ± 6.3 b
A7	Ethyl 3-methylbutanoate monomer	C_7_H_14_O_2_	130.2	1057.4	424.8	1.64903	163.9 ± 19.7 f	421.5 ± 7.0 de	489.9 ± 31.2 cd	546.8 ± 66.0 c	368.0 ± 16.0 e	706.7 ± 16.0 b	773.3 ± 41.7 b	1121.7 ± 77.2 a
A8	Ethyl 3-methylbutanoate dimer	C_7_H_14_O_2_	130.2	1058.3	425.8	1.2513	28.7 ± 4.2 e	62.9 ± 4.1 de	85.4 ± 5.8 d	136.9 ± 21.4 c	53.1 ± 5.1 de	209.8 ± 4.6 b	244.6 ± 3.3 b	755.3 ± 80.2 a
A9	Ethyl acetate	C_4_H_8_O_2_	88.1	910.2	299.1	1.33593	23535.4 ± 411.4 d	33202.4 ± 55.8 a	33147.4 ± 154.3 a	33269.6 ± 239.9 a	28010.6 ± 208.9 c	32327.3 ± 76.1 b	32943.5 ± 179.6 a	33305.2 ± 44.2 a
A10	Ethyl butanoate monomer	C_6_H_12_O_2_	116.2	1044.5	410.3	1.55569	2989.8 ± 12.7 c	3240.4 ± 20.4 a	3153.6 ± 50.6 b	2999.5 ± 65.5 c	2955.9 ± 21.8 cd	3027.9 ± 7.4 c	2968.3 ± 40.4 cd	2902.6 ± 51.1 d
A11	Ethyl butanoate dimer	C_6_H_12_O_2_	116.2	1043.6	409.3	1.20181	681.6 ± 94.9 c	1658.2 ± 66.6 b	2231.5 ± 41.0 a	2061.9 ± 294.9 a	905.8 ± 55.1 c	1734.0 ± 26.8 b	1996.6 ± 58.6 a	2213.0 ± 209.3 a
A12	Ethyl hexanoate monomer	C_8_H_16_O_2_	144.2	1239.3	706.4	1.80055	1314.1 ± 57.5 b	1556.5 ± 35.9 a	1632.6 ± 49.1 a	1408.2 ± 174.7 b	1028.9 ± 72.6 c	1068.3 ± 11.9 c	1115.8 ± 58.5 c	972.0 ± 77.3 c
A13	Ethyl hexanoate dimer	C_8_H_16_O_2_	144.2	1,243	711.8	1.34374	871.8 ± 93.6 cd	2156.1 ± 81.7 b	2551.2 ± 215.0 a	1891.9 ± 377.1 b	609.9 ± 48.1 d	831.3 ± 42.9 cd	1086.0 ± 49.8 c	804.8 ± 111.9 cd
A14	Ethyl lactate monomer	C_5_H_10_O_3_	118.1	1359.7	899.3	1.53874	8342.5 ± 3177.4 c	17552.0 ± 635.0 a	18296.3 ± 95.9 a	18008.4 ± 411.8 a	11173.3 ± 4026.6 bc	12413.7 ± 587.7 b	16830.3 ± 352.5 a	17220.5 ± 200.4 a
A15	Ethyl lactate dimer	C_5_H_10_O_3_	118.1	1,361	901.7	1.14697	3033.7 ± 2363.7 c	27066.4 ± 5344.0 b	42081.4 ± 4162.4 a	43065.2 ± 5043.2 a	7613.0 ± 7419.6 c	6602.1 ± 1007.3 c	23390.3 ± 4347.6 b	24622.7 ± 2386.7 b
A16	Ethyl propanoate	C_5_H_10_O_2_	102.1	965.4	336.7	1.45341	526.9 ± 76.9 g	1469.4 ± 35.4 e	1709.2 ± 17.4 d	1829.9 ± 109.1 c	672.1 ± 13.9 f	1535.3 ± 29.0 e	2480.9 ± 39.5 a	2290.3 ± 74.5 b
A17	Hexyl butanoate	C_10_H_20_O_2_	172.3	1468.1	1087.9	1.48649	446.4 ± 27.2 cd	690.6 ± 24.7 a	722.2 ± 24.0 a	648.3 ± 20.8 b	438.0 ± 11.1 cd	474.6 ± 15.8 c	469.2 ± 22.1 c	412.2 ± 7.5 d
A18	Isoamyl acetate monomer	C_7_H_14_O_2_	130.2	1130.9	529.6	1.74269	2106.9 ± 56.4 b	1722.8 ± 34.1 de	1711.7 ± 49.8 e	1760.4 ± 70.3 de	2207.9 ± 27.9 a	1871.3 ± 24.8 c	1790.5 ± 9.7 d	1727.8 ± 13.1 de
A19	Isoamyl acetate dimer	C_7_H_14_O_2_	130.2	1131.5	530.7	1.30775	5303.5 ± 386.3 e	8538.7 ± 205.3 abc	9207.5 ± 382.8 a	8708.8 ± 662.4 abc	7131.5 ± 256.4 d	8085.5 ± 58.8 b	8282.5 ± 156.9 bc	8960.8 ± 524.2 ab
A20	Isobutyl acetate monomer	C_6_H_12_O_2_	116.2	1022.3	385.4	1.61121	450.2 ± 25.6 c	363.0 ± 15.8 de	343.7 ± 25.9 e	335.1 ± 30.4 e	584.5 ± 19.6 a	506.4 ± 22.7 b	461.3 ± 4.5 bc	402.1 ± 45.5 d
A21	Isobutyl acetate dimer	C_6_H_12_O_2_	116.2	1020.8	383.7	1.28805	1193.6 ± 97.9 e	3158.8 ± 46.9 bc	2975.6 ± 100.5 c	3179.5 ± 186.1 b	1956.6 ± 20.6 d	3073.8 ± 52.0 bc	2970.9 ± 51.8 c	3952.7 ± 140.6 a
A22	Propyl acetate	C_5_H_10_O_2_	102.1	987.7	351.0	1.48043	666.8 ± 51.5 f	1477.2 ± 20.5 c	1606.6 ± 44.5 b	1476.5 ± 70.2 c	1129.7 ± 28.9 e	1553.7 ± 23.6 b	1307.7 ± 15.6 d	1677.9 ± 26.0 a
B1	2-butanol	C_4_H_10_O	74.1	614.1	343.9	1.56275	285.1 ± 36.9 g	787.7 ± 21.9 e	963.8 ± 10.6 d	1392.1 ± 113.8 c	472.7 ± 9.3 f	1019.7 ± 30.5 d	1794.0 ± 31.9 b	2621.9 ± 99.9 a
B2	1-propanethiol	C_3_H_8_S	76.2	603.5	228.1	1.13359	269.9 ± 9.8 f	418.4 ± 11.0 d	352.3 ± 9.0 e	474.5 ± 18.6 c	354.9 ± 22.8 e	652.3 ± 41.2 a	636.6 ± 37.8 ab	600.3 ± 43.3 b
B3	2-Methyl-1-propanol monomer	C_4_H_10_O	74.1	1100.9	476.1	1.3687	1626.4 ± 40.7 a	966.0 ± 15.7 d	990.3 ± 70.1 cd	966.0 ± 43.0 d	1494.6 ± 28.0 b	1056.2 ± 18.7 c	1000.3 ± 43.9 cd	956.8 ± 42.1 d
B4	2-Methyl-1-propanol dimer	C_4_H_10_O	74.1	1100.9	476.1	1.17201	10231.5 ± 180.4 ab	10184.5 ± 177.0 ab	9723.4 ± 274.6 cd	9269.4 ± 378.4 d	9555.2 ± 72.5 de	9946.2 ± 46.3 bc	9320.1 ± 45.4 d	10318.7 ± 139.8 a
B5	3-Methyl-1-butanol monomer	C_5_H_12_O	88.1	1216.3	673.4	1.49626	3307.2 ± 104.4 a	2678.8 ± 25.7 c	2614.8 ± 70.7 c	2594.4 ± 43.7 c	3067.1 ± 34.3 b	2679.7 ± 17.6 c	2611.6 ± 7.2 c	2625.7 ± 19.5 c
B6	3-Methyl-1-butanol dimer	C_5_H_12_O	88.1	1217.3	674.9	1.24711	12573.4 ± 219.6 d	14259.6 ± 21.9 a	14051.7 ± 286.7 ab	13265.2 ± 838.6 c	12624.5 ± 48.0 d	13202.4 ± 57.8 cd	13446.7 ± 226.1 bc	13585.5 ± 295.8 bc
B7	Butanol monomer	C_4_H_10_O	74.1	1148.8	561.5	1.38429	611.1 ± 26.3 a	306.5 ± 9.7 b	300.5 ± 5.9 bc	285.3 ± 47.5 bcd	259.1 ± 4.4 cde	245.9 ± 8.0 de	226.7 ± 9.1 e	245.6 ± 40.5 de
B8	Butanol dimer	C_4_H_10_O	74.1	1150.6	564.7	1.18309	327.3 ± 7.0 a	188.6 ± 4.9 bc	220.6 ± 23.9 b	155.3 ± 55.3 c	49.3 ± 2.7 d	72.5 ± 5.1 d	63.8 ± 2.1 d	83.8 ± 7.9 d
B9	Ethanol	C_2_H_6_O	46.1	945.3	322.6	1.14763	21165.5 ± 105.7 cd	22572.7 ± 24.9 a	22591.0 ± 210.8 a	21670.0 ± 480.7 b	20839.5 ± 134.7 d	21546.1 ± 111.3 bc	21498.0 ± 59.7 bc	21863.5 ± 255.6 b
C1	2-methyloctane	C_9_H_20_	128.3	858	264.2	1.0634	1216.1 ± 42.9 g	2547.4 ± 38.4 e	2573.2 ± 76.9 de	2669.6 ± 49.8 c	2130.4 ± 40.3 f	2646.5 ± 31.5 cd	2753.6 ± 27.3 b	3081.7 ± 22.8 a
C2	Oct-2-ene	C_8_H_16_	112.2	821.3	239.6	1.08438	278.2 ± 13.0 d	152.9 ± 9.5 e	140.2 ± 5.8 e	134.3 ± 12.3 e	507.9 ± 13.2 a	291.3 ± 12.5 cd	373.0 ± 4.8 b	341.2 ± 78.7 bc
C3	1,1,1-Trichloroethane	C_2_H_3_Cl_3_	133.4	658	264.2	1.0634	278.5 ± 14.9 d	265.6 ± 2.2 de	241.9 ± 2.9 f	254.7 ± 19.7 ef	315.5 ± 12.1 c	367.2 ± 4.6 b	440.8 ± 7.7 a	315.3 ± 22.2 c
C4	2-Pentenal	C_5_H_8_O	84.1	746.8	393.7	1.32854	203.9 ± 4.6 f	580.6 ± 15.6 d	667.7 ± 30.3 c	620.8 ± 39.4 d	482.0 ± 6.3 e	751.2 ± 12.8 b	703.8 ± 8.0 c	827.2 ± 52.3 a
D1	2-methylfuran	C_5_H_6_O	82.1	612.3	251.0	1.06259	1583.7 ± 126.8 a	165.6 ± 11.9 c	183.4 ± 5.2 c	228.8 ± 8.6 c	1531.4 ± 47.4 a	242.6 ± 5.4 c	350.6 ± 7.5 b	230.4 ± 45.5 c
D2	Tetrahydrofuran	C_4_H_8_O	72.1	629.3	239.6	1.08438	1451.3 ± 89.6 a	255.8 ± 58.9 c	286.8 ± 18.0 c	325.2 ± 12.5 c	1250.4 ± 14.9 b	272.7 ± 40.3 c	331.2 ± 15.4 c	267.7 ± 105.9 c
D3	Thiophene	C_4_H_4_S	84.1	686.8	350.5	1.27262	91.6 ± 12.8e	289.3 ± 10.5 d	350.7 ± 8.1 bc	389.6 ± 55.3 b	135.1 ± 2.3 e	336.1 ± 7.1 c	559.2 ± 16.1 a	577.4 ± 41.7 a
E1	4-methylpentanal	C_6_H_12_O	100.2	1030.2	391.8	1.45233	38.4 ± 7.4 f	171.4 ± 1.8 e	217.3 ± 11.7 d	204.9 ± 32.3 d	201.3 ± 5.3 d	339.6 ± 6.9 b	289.3 ± 9.2 c	386.8 ± 14.1 a
E2	Butanal monomer	C_4_H_8_O	72.1	849.8	258.7	1.27998	1115.7 ± 30.6 c	940.7 ± 16.7 d	822.2 ± 16.9 e	812.3 ± 17.4 e	1111.5 ± 8.6 c	1234.5 ± 13.8 b	1533.9 ± 18.7 a	1135.2 ± 66.2 c
E3	Butanal dimer	C_4_H_8_O	72.1	852	260.2	1.10766	1172.8 ± 113.8 cd	1120.3 ± 52.1 de	1264.9 ± 32.0 bc	1279.2 ± 15.2 bc	1873.7 ± 87.1 a	738.1 ± 37.3 e	1035.0 ± 42.6 d	1366.5 ± 50.5 b
F1	3-hydroxy-2-butanone dimer	C_4_H_8_O_2_	88.1	1294.9	786.8	1.07149	12871.4 ± 1184.7 a	2996.5 ± 2714.6 b	386.1 ± 61.6 c	269.1 ± 49.c8	3188.9 ± 508.9 a	519.4 ± 273. 6 c	321.5 ± 43.5 c	263.4 ± 20.8 c
F2	3-Hydroxy-2-butanone monomer	C_4_H_8_O_2_	88.1	1295.6	787.9	1.33247	5490.7 ± 240. 3 a	2401.5 ± 913.0 c	1059.7 ± 136.5 d	881.0 ± 31.3d	3170.9 ± 283.7 b	1242.5 ± 286.6 d	980.8 ± 23.6 d	822.3 ± 6.4 d
G1	Acetic acid monomer	C_2_H_4_O_2_	60.1	1,524	1185.2	1.15805	11809.5 ± 318.2 c	12005.7 ± 446.5 bc	12339.4 ± 132.1 ab	12245.5 ± 143.1 abc	12657.1 ± 125.8 a	12293.4 ± 245.4 abc	12091.3 ± 370.7 bc	11948.2 ± 179.0 bc
G2	Acetic acid dimer	C_2_H_4_O_2_	60.1	1525.3	1187.3	1.05271	4621.6 ± 364.1 e	7557.9 ± 2100.9 cd	10501.9 ± 1146.4 a	8592.5 ± 1152.8 b	5219.5 ± 198.5 de	5608.8 ± 543.5 cde	6692.4 ± 1167.4 bcd	6855.3 ± 597.3 bcd

All data are presented as mean ± standard deviation (*n* = 3). Values with different letters in a row indicate significant differences at *p* < 0.05 as determined by one-way ANOVA Duncan’s test.

**Figure 6:**
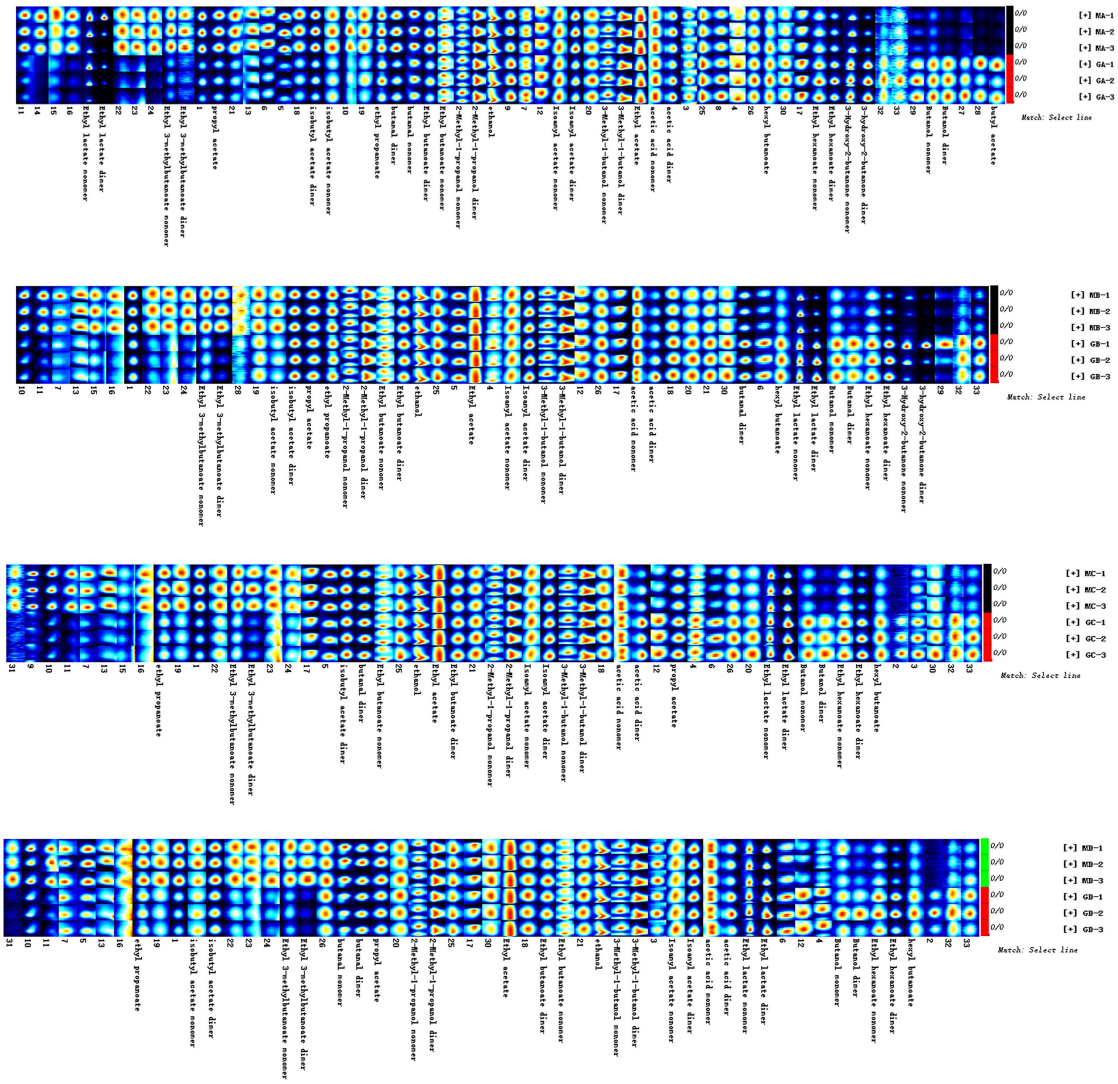
Gallery plot of the volatile flavor compounds based on the gas phase ion mobility spectrum. The numbers represent unidentified compounds in the mobility library.

As shown in Figure 6, main flavor compound composition and abundance varied not only between MA–MD samples but also significantly varied between MA–MD samples and samples GA–GD. Although 46 compounds were identified across all samples, their abundances differed. In MA samples, 18 compounds showed significantly higher abundances as compared to their levels in GA samples, with compounds such as 2-butanol, oct-2-ene, 1-methylethyl acetate, tert-butyl acetate, propyl propanoate, 2-pentenal, and 4-methylpentanal being particularly prominent (Figure 6). However, the abundance of the 2-methyl-1-propanol monomer, the 3-hydroxy-2-butanone monomer, the 3-methyl-1-butanol monomer, the butanol dimer, the butanol monomer, and butyl acetate was higher in GA samples than in MA samples. Eleven compounds, i.e., the ethyl 3-methylbutanoate dimer, the ethyl 3-methylbutanoate monomer, 1-propanethiol, oct-2-ene, 1,1,1-trichloroethane, 1-methylethyl acetate, tert-butyl acetate, 2-butanol, propyl propanoate, 2-pentenal, and 4-methylpentanal, exhibited significantly higher abundances in MB samples than in GB samples; however, the abundance of the ethyl hexanoate dimer, ethyl hexanoate monomer, ethyl lactate dimer, butanol dimer, butanol monomer, 3-hydroxy-2-butanone dimer, and 3-hydroxy-2-butanone monomer was higher in GB samples than in MB samples. The abundance of the ethyl 3-methylbutanoate dimer, the ethyl 3-methylbutanoate monomer, ethyl propanoate, 2-methylpropyl propionate, 2-methylfuran, 1-propanethiol, oct-2-ene, 1,1,1-trichloroethane, 1-methylethyl acetate, thiophene, and 2-butanol was significantly higher in GC samples than in GA samples; however the abundance of hexyl butanoate, the ethyl hexanoate dimer, the ethyl hexanoate monomer, the butanol dimer, and the butanol monomer was significantly lower in GC samples than in GA samples. The abundance of the ethyl 3-methylbutanoate dimer, the ethyl 3-methylbutanoate monomer, ethyl propanoate, 2-butanol, 2-methyloctane, 1-propanethiol, oct-2-ene, thiophene, propyl propanoate, 2-pentenal, 4-methylpentanal, and 2-methylpropyl propionate was significantly higher in MD samples than in GD samples; however, the abundance of hexyl butanoate, the ethyl hexanoate dimer, the ethyl hexanoate monomer, the butanol dimer, and the butanol monomer was significantly lower in MD samples than in GD (Figure 6; Table 2).

**Figure 7:**
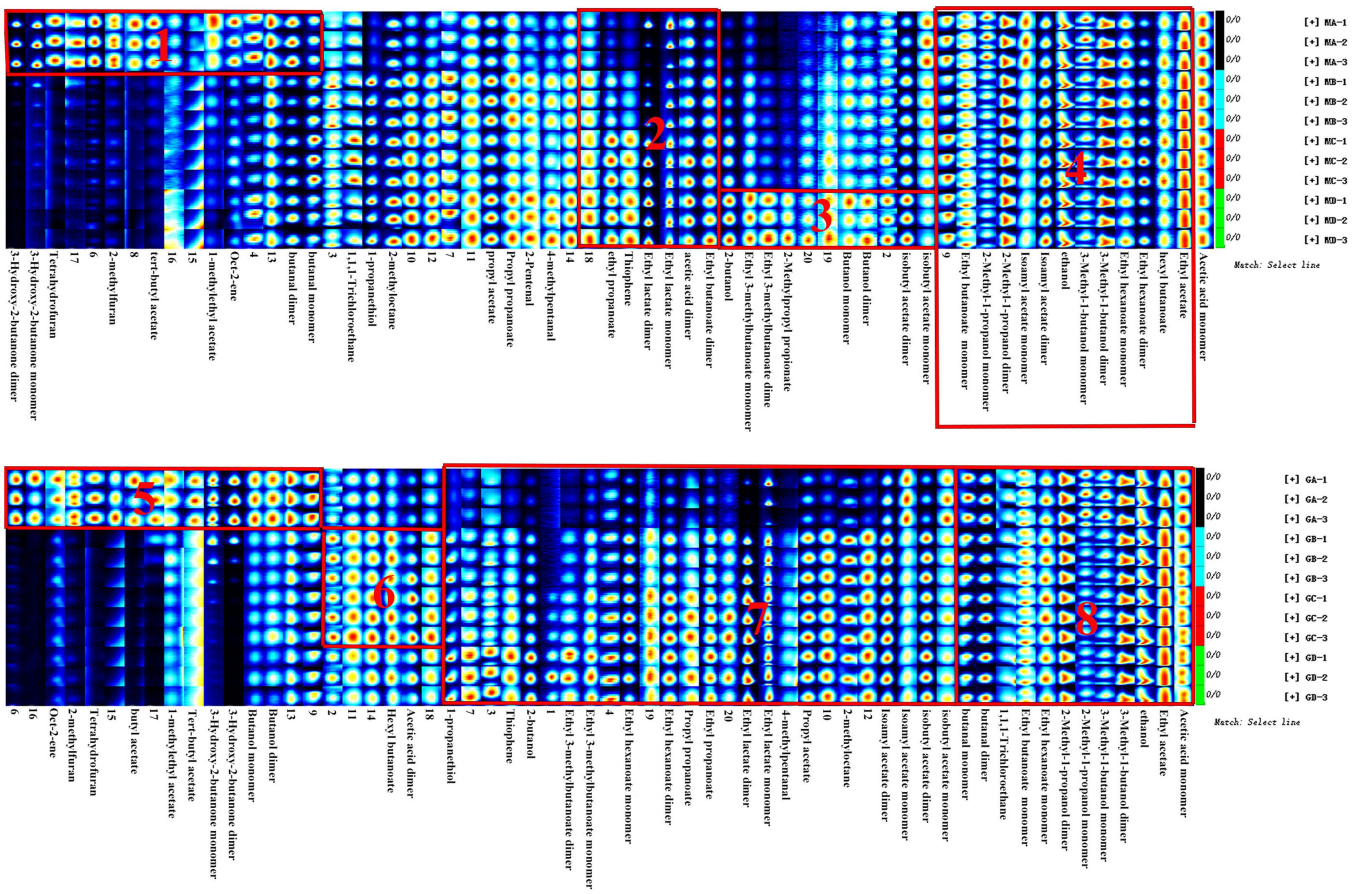
Gallery plot of volatile flavor compounds based on the gas phase ion mobility spectrum at different stages of proso millet and sorghum fermentation. The numbers represent unidentified compounds in the mobility library.

In addition, as shown in Figure 7, the abundance of compounds in areas 1 and 3 was higher than that in other samples, and these compounds could be used as characteristic flavor compounds in MA and MD samples, respectively. In area 2, the levels of compounds such as ethyl propionate, ethyl lactate, acetic acid, and ethyl butyrate gradually increased and attained a maximum abundance during fermentation. In area 4, compound abundance remained relatively constant during fermentation. Flavor compounds in MA and MD samples were significantly different, whereas those in MB and MC samples were relatively similar. In area 5, the abundance of compounds such as butyl acetate, 3-hydroxy-2-butanone, and butyraldehyde was significantly higher in GA samples than in GB–GD samples. In area 6, compound abundance initially increased and then decreased. However, in area 7, the abundance of compounds such as ethyl 3-methylbutyrate, ethyl caproate, ethyl butyrate, ethyl propionate, ethyl lactate, propyl acetate, isoamyl acetate, and isobutyl acetate, gradually increased during fermentation. In area 8, compound abundance remained relatively constant throughout the fermentation process.

### 3.3 Relationship between microbial composition changes and flavor compound formation

**Figure 8:**
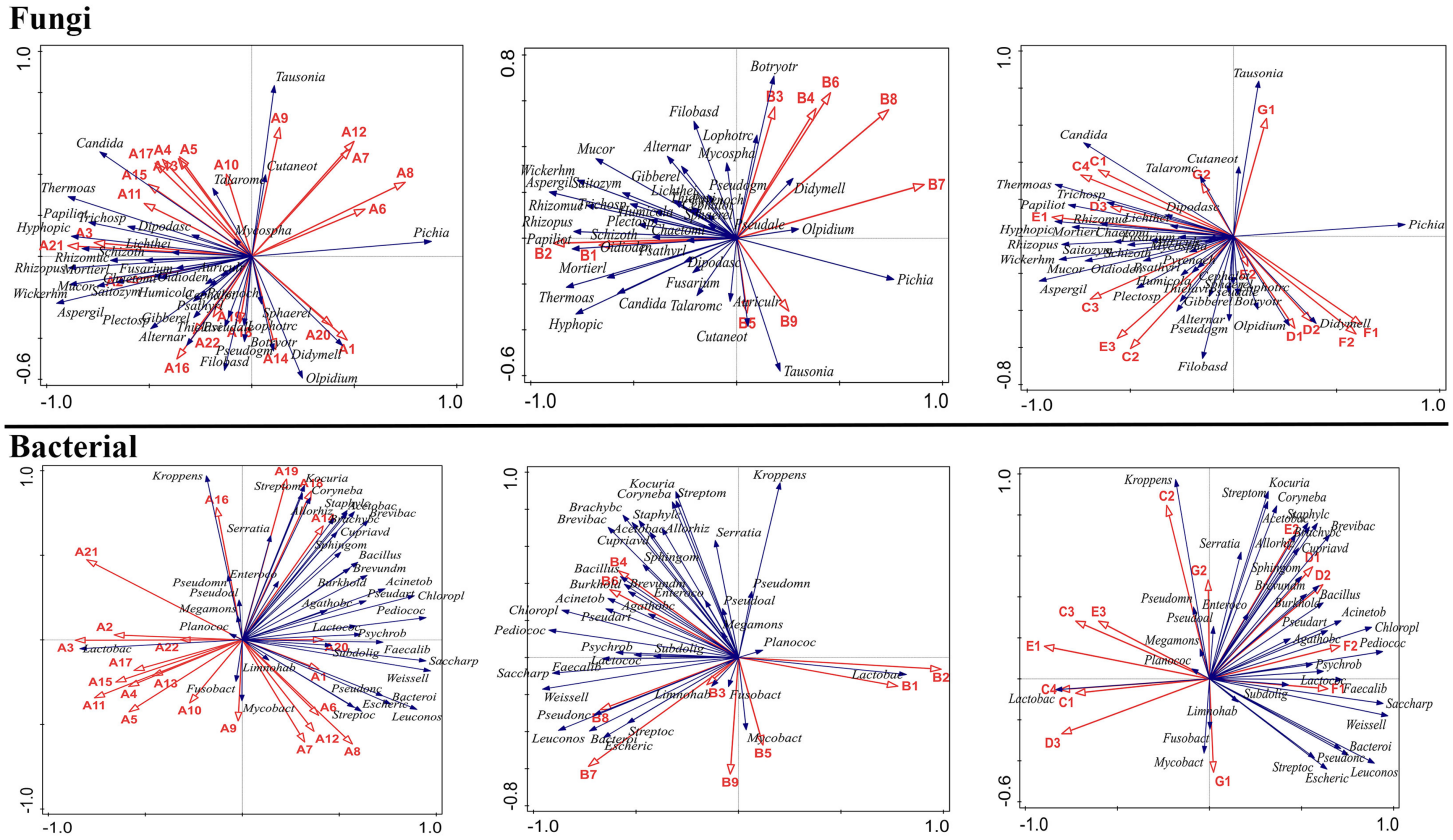
Relationship between 45 flavor compounds and microbial communities at the genus level.

**Figure 9:**
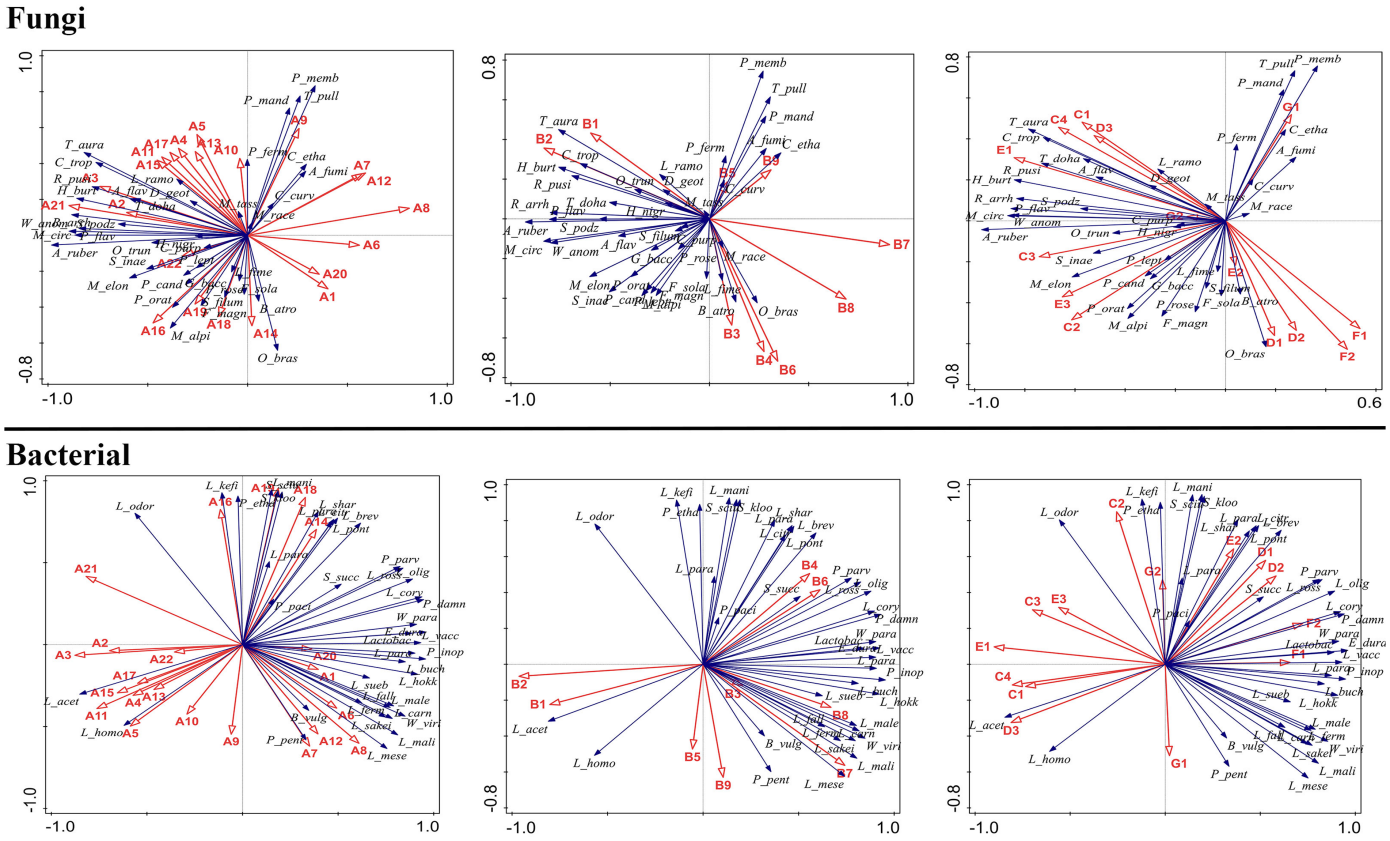
Relationship between 45 flavor compounds and microbial communities at the species level.

The RDA analysis result showed that bacteria from the *Weissella*, *Acinetobacter*, *Bacteroides*, *Psychrobacter*, *Pseudarthrobacter*, *Lactococcus*, *Chloroplast*, *Saccharopolyspora*, *Psychrobacter*, *Saccharopolyspora*, *Pseudonocardiaceae*, and *Bacteroides* genera and fungi from the *Thermoascus*, *Aspergillus*, *Pichia*, *Rhizomucor*, *Papiliotrema*, *Hyphopichia*, and *Mucor* genera significantly inhibited the synthesis of the flavor compounds, such as ethyl hexanoate, ethyl butanoate, ethyl lactate ethyl lactate, and butyl acetate, but improved the abundance of ethyl acetate (*p* < 0.05) (Figures 8, 9). Moreover, these microbes exhibited a greater abundance in proso millet–sorghum mixed fermentation samples than in sorghum samples. The synthesis of special flavor compounds in proso millet Baijiu was significantly promoted in the presence of fungi from the *Rhizopus*, *Papiliotrema*, *Wickerhamomyces*, *Aspergillus*, and *Thermoascus* genera but inhibited by bacteria from the *Weissella*, *Acinetobacter*, *Psychrobacter*, *Pseudarthrobacter*, *Bacteroides*, and *Saccharopolyspora* genera (Figures 8, 9).

Among the 40 fungal and bacterial species with the highest abundance, all bacterial and 30 fungal species were found to be significantly associated with changes in the abundance of flavor compounds (Supplementary Table S2). More specifically, among these 30 fungal species, *P. fermentans*, *C. tropicalis*, *D. geotrichum*, *S. inaequale*, *P. membranifaciens*, *C. curvatus*, *C. ethanolica*, and *R. arrhizus* played significant roles in altering the abundance of flavor compounds, and were significantly correlated with changes in the abundance of 18, 18, 18, 15, 13, 11, 10, and 9 types of flavor compounds, respectively. In addition, eight main fungal species exerted a significant effect on changes in the abundance of 41 compounds, with the exception of the ethyl 3-methyl butanoate monomer, the ethyl 3-methyl butanoate dimer, 2-methylfuran, and thiophene. The ethyl 3-methyl butanoate monomer and the ethyl 3-methyl butanoate dimer were found to be significantly positively correlated with the presence of *F. solani* and *P. candolleana* (*p* < 0.05). In addition, the abundance of 2-methylfuran was significantly positively correlated with *F. solani* abundance (correlation coefficient *R* = 0.475), but negatively correlated with *R. arrhizus* abundance (correlation coefficient *R* = −0.443) (*p* < 0.05). Thiophene abundance was significantly positively correlated with *P. fermentans*, *T. pullulans*, and *C. ethanolica* abundance (*p* < 0.01), with correlation coefficients of 0.701, 0.600, and 0.741, respectively; however, thiopene abundance was negatively correlated with *G. baccata* abundance (*p* < 0.05) (correlation coefficient *R* = −0.431).

Among the 40 most significant bacterial species, *L. oligofermentans*, *L. acetotolerans*, *L. coryniformis*, *W. paramesenteroides*, *L. mali*, *E. durans*, *L. paralimentarius*, *L. vaccinostercus*, *L. mesenteroides*, *L. homohiochii*, *L. fallax*, *W. viridescens*, *L. fermentum*, *L. suebicus*, *L. sakei*, and *L. citreum* were the main species the abundance of which was significantly correlated with changes in the abundance of flavor compounds, with these species inducing significant changes in the abundance of 31, 30, 30, 29, 29, 29, 28, 28, 28, 27, 27, 27, 26, 26, 25, and 25 types of flavor compounds, respectively. Fifteen of these main bacterial species exerted significant positive or negative effects on 44 flavor compounds, except for 2-methylpropyl propionate. Furthermore, the abundance of 2-methylpropyl propionate was significantly negatively correlated with the presence of *L. buchneri* (*R* = −0.406; *p < 0.05*). Furthermore, the formation of this flavor compound was significantly associated with the presence of the fungal species, *F. solani*, *P. candolleana*, *M. elongat*, *S. inaequale*, and *P. leptospora*, with correlation coefficients of 0.631 (*p* < 0.01), 0.741 (*p* < 0.01), 0.512 (*p* < 0.05), 0.690 (*p* < 0.01), and 0.453 (*p* < 0.05), respectively.

Furthermore, based on a redundancy analysis and Pearson’s correlation coefficients (Figures 8, 9), we found that the relative abundance of esters was significantly correlated with the presence of fungal species such as *P. fermentans*, *A. ruber*, *C. tropicalis*, *R. arrhizus*, *A. flavus*, *P. membranifaciens*, *O. brassicae*, *M. circinelloides*, *F. solani*, *B. atrogriseum*, *S. podzolica*, *T. dohaense*, *H. nigrescens*, *T. pullulans*, *C. ethanolica*, *P. candolleana*, *D. geotrichum*, *M. elongata*, *G. baccata*, *F. magnum*, *A. fumigatus*, *C. curvatus*, *O. truncatum*, *L. fimeti*, and *S. inaequale*. In addition, the abundance of esters was also correlated with the presence of 38 bacterial species, excluding *L. parafarraginis* and *P. pacificensis*. Changes in the relative abundance of alcohols were significantly correlated with the presence of several fungal species, including *P. fermentans*, *A. ruber*, *C. tropicalis*, *R. arrhizus*, *A. flavus*, *P. membranifaciens*, *O. brassicae*, *F. solani*, *B. atrogriseum*, *S. podzolica*, *C. ethanolica*, *P. candolleana*, *D. geotrichum*, *P. flavescens*, *F. magnum*, *C. curvatus*, *O. truncatum*, and *S. inaequale*. Changes in alcohol abundance were also correlated with the presence of 37 bacterial species, excluding *L. parafarraginis*, *P. pacificensis*, and *S. succinus*. Hydrocarbon abundance was significantly correlated with the presence of specific fungal species, including *P. fermentans*, *C. tropicalis*, *R. arrhizus*, *A. flavus*, *P. membranifaciens*, *O. brassicae*, *B. atrogriseum*, *T. pullulans*, *C. ethanolica*, *G. baccata*, and *C. curvatus*. In addition, hydrocarbon abundance was correlated with the presence of 37 bacterial species, excluding *L. parafarraginis*, *P. pacificensis*, and *S. succinus*. Heterocyclic compound abundance was significantly correlated with the presence of the fungal species, *D. geotrichum* and *S. inaequale*, as well as that of 34 bacterial species, excluding *L. hokkaidonensis*, *L. parafarraginis*, *P. pacificensis*, *S. succinus*, *P. pentosaceus*, and *B. vulgatus*. Aldehyde abundance was significantly correlated with the presence of fungal species such as *P. fermentans*, *T. aurantiacus*, *R. arrhizus*, *P. membranifaciens*, *F. solani*, *M. tassiana*, *T. dohaense*, *H. nigrescens*, *T. pullulans*, *C. ethanolica, G. baccata*, and *C. curvatus*. In addition, aldehyde abundance was correlated with the presence of 36 bacterial species, excluding *L. parafarraginis*, *P. pacificensis*, *L. homohiochii*, and *B. vulgatus*. Ketone abundance was correlated with the presence of the fungal species, *C. tropicalis*, *R. arrhizus*, *S. podzolica*, *D. geotrichum*, and *S. inaequale*, as well as that of bacterial species such as *L. acetotolerans*, *P. parvulus*, *L. mesenteroides*, *L. coryniformis*, *P. damnosus*, *L. homohiochii*, *W. viridescens*, *L. oligofermentans*, *L. rossiae*, *L. paralimentarius*, *W. paramesenteroides*, *L. vaccinostercus*, *L. sakei*, *L. mali*, *E. durans*, *L. fermentum*, *Lactobacillus* sp., *L. sharpeae*, *L. citreum*, *L. suebicus*, and *L. fallax*. Organic acid abundance was correlated with the presence of fungal species such as *P. fermentans*, *C. tropicalis*, *P. membranifaciens*, *L. ramosa*, *M. circinelloides*, *S. podzolica*, *H. nigrescens*, *G. baccata*, and *A. fumigatus*, as well as that of bacterial species such as *L. brevis*, *L. parabuchneri*, *P. inopinatus*, *L. kefiri*, *L. homohiochii*, *W. viridescens*, *L. malefermentans*, *L. manihotivorans*, *L. odoratitofui*, *L. oligofermentans*, *L. rossiae*, *L. hokkaidonensis*, *L. pontis*, *S. kloosii*, *P. ethanolidurans*, *L. mali*, *P. pentosaceus*, *L. fermentum*, *S. sciuri*, *L. sharpeae*, *L. citreum*, *L. suebicus*, and *L. fallax*.

## 4 Discussion

Proso millet, a small miscellaneous grain rich in β-carotene and multiple vitamins, is often utilized in the production of rice wine and other viscous foods due to its high viscosity. However, its application in Chinese Baijiu fermentation faces significant challenges. In a previous study, to effectively utilize millet in Chinese Baijiu fermentation, we optimized the fermentation process and successfully developed a new Chinese Baijiu product. Moreover, sensory evaluation indicated significant differences in taste and flavor between the new Chinese Baijiu product and traditional variants. Therefore, this study aimed to describe differences between traditional and new Chinese Baijiu products through the analysis of microbial communities and flavor at various fermentation stages.

### 4.1 Changes in core microbial communities in proso millet Chinese Baijiu at different fermentation stages

Chinese Baijiu is produced through microbial fermentation and its quality is related to the microbial communities involved in its production. Analysis of changes in microbial communities revealed that the core microbial communities in samples MA–MD differed from those in samples GA–GD. Thus, proso millet may be an effective material for improving the quality of Baijiu. Compared to sorghum samples, proso millet samples exhibited a significant increase in microbial diversity during fermentation, as well as a change in the core microbial community composition. Among the core microbes involved in proso millet Baijiu production, only fungi of the genera, *Mucor*, *Thermoascus*, *Aspergillus*, *Rhizomucor*, *Rhizopus*, *Pichia*, and *Candida* and bacteria of the genera, *Bacillus*, *Lactobacillus*, *Lactococcus*, *Weissella*, *Staphylococcus*, *Saccharopolyspora*, *Streptococcus*, and *Pediococcus*, have been previously reported to be involved in traditional Chinese Baijiu production (Yang et al., 2017; Zou et al., 2018; Gan et al., 2019; Xie et al., 2020). At the species level, aside from the fungal species, *L. ramosa*, *C. tropicalis*, *A. flavus*, *M. racemosus*, and *W. anomalus*, and the bacterial species, *L. brevis*, *L. buchneri*, *P. parvulus*, *L. odoratitofui*, *L. citreum*, *Lactobacillus* sp., *L. pontis*, *P. pentosaceus*, and *L. homohiochii*, the rest of the core microbes identified in our study have not been reported in other traditional Chinese Baijiu beverages (Huang et al., 2020; Sakandar et al., 2020; Wu et al., 2021; Xie et al., 2021).

In addition, the core microbes identified at different stages of proso millet Chinese Baijiu fermentation in this study are different from those identified in other traditional Chinese Baijiu beverages. For instance, for Xiaoqu Baijiu (Jing Brand Co., Ltd.), core microbes identified at the beginning of the fermentation process included *Leuconostoc lactis*, *W. paramesenteroides*, *Lactococcus lactis*, *Lactobacillus delbrueckii*, *Gluconacetobacter sacchari*, *Rhizopus oryzae*, and *Hypocreales* sp., and those identified at the end of the fermentation process included *Acetobacter pasteurianus*, *L. pontis*, *L. acetotolerans*, *W. anomalus*, *Monascus purpureus*, *C. tropicalis*, and *A. flavus* (Xie et al., 2021). Similarly, in light-flavored Daqu Baijiu (Xinghuacun Fenjiu Distillery Co., Ltd.), the core microbes shifted from species such as *P. kudravzerii*, *L. ramosa*, *L. citreum*, and *B. licheniformis* on day 1 of fermentation to *L. acetotolerans*, *L. buchneri*, and *L. hilgardii* at the end of fermentation (Huang et al., 2020). In Chinese strong-flavored Baijiu, the main microbes identified during the early stages of fermentation (days 1–23) included *Lactobacillus*, *Thermoascus*, *Aspergillus*, *Emericella*, *Monascus*, *Candida,* unidentified *Saccharomycetales*, *Rhizopus*, and *Pichia* species; however, during the mid-stage of fermentation (days 23–48), *Thermoascus*, *Aspergillus*, *Emericella*, *Candida*, and unidentified *Saccharomycetales* species were the primary microbes identified (Zhang et al., 2017). In MA samples, at the beginning of fermentation, the main core fungal species identified included *F. solani*, *M. elongata*, *P. oratosquillae*, *P. leptospora*, *P. candolleana*, and *S. inaequale* (abundance >1%), and the primary bacterial species identified included *L. vaccinostercus*, *L. mesenteroides*, *L. mali*, *L. suebicus*, *L. sakei*, *L. fallax*, *W. viridescens*, *L. fermentum*, *L. paralimentarius*, and *W. paramesenteroides*. The core microbes identified in MB and MC samples during mid-stage fermentation included *S. inaequale*, *H. nigrescens*, *D. geotrichum*, *C. tropicalis*, *T. dohaense*, *M. circinelloides*, *S. podzolica*, *P. roseus*, *G.*, *L. ramose*, *S. filum*, *O. truncatum*, *L. parafarraginis*, *L. buchneri*, *B. vulgatus*, *L. malefermentans*, *L. carnosum*, *P. inopinatus*, and *L. hokkaidonensis*, and core microbes identified in MD samples during the end of fermentation included *C. purpureofuscum*, *F. magnum*, *A. ruber*, *R. pusillus*, *A. flavus*, *R. arrhizus*, *P. flavescens*, *W. anomalus*, *L. homohiochii*, and *P. pentosaceus* (Figures 2, 3). These differences in core microbial communities across different Baijiu products highlight the impact of fermentation raw materials, technology, and *Daqu* on microbial communities.

### 4.2 Flavor compound formation in proso millet Chinese Baijiu and differences between proso millet Chinese Baijiu and other products

Compared to sorghum fermentation, proso millet fermentation induced a change in the core flavor compounds of traditional light-flavored Baijiu from ethyl acetate, ethyl hexanoate, ethyl hexanoate dimer, ethyl butanoate, ethyl lactate, and butyl acetate to oct-2-ene, 2-butanol, propyl propanoate, 2-pentenal, and 4-methylpentanal. However, further evaluation is required to determine the contribution of each volatile flavor compound to the overall flavor of Baijiu. In this study, we detected a total of 22 esters, 9 alcohols, 4 hydrocarbons, 3 heterocyclic compounds, 3 aldehydes, 2 ketones, and 2 organic acids in proso millet Chinese Baijiu through GC-IMS. Based on their relative peak areas (above 1800), the key flavor compounds in proso millet Chinese Baijiu were identified to be ethyl acetate (A9), ethyl butanoate dimer (A11), ethyl lactate monomer (A14), ethyl lactate dimer (A15), isoamyl acetate monomer (A18), isoamyl acetate dimer (A19), isobutyl acetate monomer (A20), 2-methyl-1-propanol monomer (B3), 3-methyl-1-butanol monomer (B5), 3-methyl-1-butanol dimer (B6), 2-methyloctane (C1), butanal monomer (E2), 3-hydroxy-2-butanone dimer (F1), 3-hydroxy-2-butanone monomer (F2), acetic acid monomer (G1), and acetic acid dimer (G2) (Figure 7; Table 2).

However, other traditional Chinese Baijiu formulas have different flavor compound compositions. For instance, strong-aroma Baijiu contains 11 main flavor compounds: ethyl hexanoate, ethyl acetate, ethyl lactate, ethyl butyrate, ethyl valerate, ethyl heptanoate, hexanoic acid, butanoic acid, heptanoic acid, furfural, and phenylethyl alcohol. Light-aroma Baijiu mainly contains 6 compounds: ethyl acetate, ethyl lactate, acetic acid, 2-methylpropanoic acid, β-damascenone, and terpenoids. Ten main flavor compounds (ethyl hexanoate, hexanoic acid, 3-methylbutanoic acid, 3-methylbutanol, tetramethylpyrazine, ethyl 2-phenylacetate, 2-phenylethyl acetate, ethyl 3-phenylpropanoate, 4-methylguaiacol, and γ-decalactone) were detected in sauce-aroma Baijiu. Ethyl acetate, ethyl caproate, and isoamyl alcohol were identified as the main flavor compounds in feng-aroma Baijiu (Liu et al., 2018; Fang et al., 2019). In addition, 1874 types of volatile flavor compounds have been identified in Chinese spirits, and Fenjiu alone is marketed in nine series and 113 products, graded by flavor and quality level. The new product, proso millet Baijiu, stands out as it contains significantly different flavor compounds as compared to other light-flavored Baijiu products; these include the ethyl butanoate dimer (A11), ethyl lactate monomer (A14), ethyl lactate dimer (A15), isoamyl acetate monomer (A18), isoamyl acetate dimer (A19), 2-methyloctane (C1), butanal monomer (E2), 3-hydroxy-2-butanone dimer (F1), and 3-hydroxy-2-butanone monomer (F2), which are not found in other Fenjiu products (Li et al., 2020). Furthermore, the relative abundance of the same flavor compounds varies between different Chinese spirits, indicating the distinct flavor characteristics of Fenjiu series products, including proso millet light-flavored Baijiu, as compared to other Baijiu formulas (Li et al., 2020; Wang J.-Y. et al., 2020). Studies have shown that raw materials, Daqu, and the fermentation process may be the primary parameters underlying flavor differences in different products. Thus, proso millet Baijiu is a new light-flavored Baijiu product and an effective material for enhancing the flavor of Baijiu. Moreover, modifying the fermentation and Daqu-making technology is an important approach for improving the flavor of Baijiu. Finally, although 45 flavor compounds were identified through GC-IMS, 20 other flavor compounds need to be further identified through alternative detection methods.

### 4.3 Relationship between core microbes and flavor compound formation

The RDA analysis showed that proso millet induced a change in the core flavor compounds of sorghum Baijiu by increasing the abundance of flavor-related microbes, such as bacteria from the *Weissella*, *Acinetobacter*, *Bacteroides*, *Psychrobacter*, *Pseudarthrobacter*, *Lactococcus*, *Chloroplast*, *Saccharopolyspora*, *Psychrobacter*, *Saccharopolyspora*, *Pseudonocardiaceae*, and *Bacteroides* genera and fungi from the *Thermoascus*, *Aspergillus*, *Pichia*, *Rhizomucor*, *Papiliotrema*, *Hyphopichia*, and *Mucor* genera, the abundance of which was significantly greater in proso millet–sorghum mixed fermentation samples than in sorghum samples. In addition, among the core fungal species, *F. solani* was significantly positively correlated with the formation of the main flavor compound, A20. Furthermore, *P. candolleana* was significantly positively correlated with the formation of the main flavor compound, B3. *S. inaequale* was significantly negatively correlated with the formation of the main flavor compound, A9, but was significantly positively correlated with the formation of the main flavor compounds, B3, B4, B6, F1, and F2. Furthermore, *T. aurantiacus* and *M. tassiana* were significantly negatively correlated with the formation of the main flavor compound, E2. In addition, *A. ruber* was significantly positively correlated with the formation of the main flavor compounds, A11 and B9. *R. arrhizus* was significantly negatively correlated with the formation of the main flavor compound, C1, but was significantly positively correlated with the formation of the main flavor compound, F1. *H. nigrescens* was significantly negatively correlated with the formation of the main flavor compounds, E2 and G2. Furthermore, *D. geotrichum* was significantly negatively correlated with the formation of the main flavor compounds, A9, A15, A18, and A20, but was significantly positively correlated with the formation of the main flavor compounds, A19, B4, B6, and F2. *C. tropicalis* was significantly negatively correlated with the formation of the main flavor compounds, A9, A14, A18, A20, C1, G1, and G2, but was significantly positively correlated with the formation of the main flavor compounds, B4, B6, F1, and F2. *T. dohaense* was significantly negatively correlated with the formation of the main flavor compounds, A15 and E2. *M. circinelloides* was significantly negatively correlated with the formation of the main flavor compounds, A14, A15, and G2. *S. podzolica* was significantly negatively correlated with the formation of the main flavor compound, G2, but was significantly positively correlated with the formation of the main flavor compound, F1. *G. baccata* and *A. fumigatus* were significantly positively correlated with the formation of the main flavor compound, G1. *L. ramosa* was significantly negatively correlated with the formation of the main flavor compound, G2. *O. truncatum* was significantly positively correlated with the formation of the main flavor compounds, B5 and B9. *F. magnum* was significantly negatively correlated with the formation of the main flavor compounds, A19 and B3. Furthermore, *B. atrogriseum* was significantly negatively correlated with the formation of the main flavor compound, B3, but was significantly positively correlated with the formation of the main flavor compound, A14. *P. fermentans* was significantly negatively correlated with the formation of the main flavor compounds, A14, B9, and G1, but was significantly positively correlated with the formation of the main flavor compounds, A19 and G2. *C. curvatus* was significantly negatively correlated with the formation of the main flavor compound, A11, but was significantly positively correlated with the formation of the main flavor compound, E2. *T. pullulans* was significantly negatively correlated with the formation of the main flavor compound, A14.

Among the core bacterial species, *L. acetotolerans* was significantly negatively correlated with the formation of the main flavor compounds, A19, B4, B6, E2, F1, and F2, but was significantly positively correlated with the formation of the main flavor compounds, A9, A15, A18, A20, and C1. *L. homohiochii* was significantly negatively correlated with the formation of the main flavor compounds, A19, B3, B4, B6, E2, F1, and F2, but was significantly positively correlated with the formation of the main flavor compounds, A9, A14, A15, A18, A20, C1, and G1. *P. pentosaceus* was significantly positively correlated with the formation of the main flavor compounds, A14, A15, B9, and G1. *P. parvulus* was significantly positively correlated with the formation of the main flavor compounds, A9, A19, A20, B4, B6, E2, and F1. *P. damnosus* was significantly negatively correlated with the formation of the main flavor compounds, A9, A19, and C1, but was significantly positively correlated with the formation of the main flavor compounds, A19, B4, B6, E2, and F2. *L. coryniformis* was significantly negatively correlated with the formation of the main flavor compounds, A9, A14, A15, A18, A20, B5, B9, and C1, but was significantly positively correlated with the formation of the main flavor compounds, A19, B4, B6, E2, F1, and F2. *L. oligofermentans* was significantly negatively correlated with the formation of the main flavor compounds, A14, A15, A18, A20, B5, B9, C1, and G1, but was significantly positively correlated with the formation of the main flavor compounds, A9, A19, B4, B6, E2, F1, and F2. *L. buchneri* and *B. vulgatus* were significantly positively correlated with the formation of the main flavor compounds, A11, B5, and B9. Furthermore, *L. malefermentans* and *L. hokkaidonensis* were significantly positively correlated with the formation of the main flavor compounds, A11, A14, B5, B9, and G1. *L. carnosum* was significantly positively correlated with the formation of the main flavor compound, A11, but was negatively correlated with the formation of the main flavor compound, C1. *P. inopinatus* was significantly positively correlated with the formation of the main flavor compounds, A11, B9, and G1. *E. durans* was significantly positively correlated with the formation of the main flavor compounds, A19, B4, B6, F1, and F2, but was negatively correlated with the formation of the main flavor compounds, A9, A18, A20, and C1. *L. vaccinostercus* and *L. mesenteroides* were significantly positively correlated with the formation of the main flavor compounds, A11, B3, B4, B6, F1, and F2, but were negatively correlated with the formation of the main flavor compounds, A9, A18, A20, and C1. *L. mali* was significantly positively correlated with the formation of the main flavor compounds, B3, B4, B6, F1, and F2, but was negatively correlated with the formation of the main flavor compounds, A9, A18, A20, C1, and G2. *L. suebicus* was significantly positively correlated with the formation of the main flavor compounds, B3, B6, F1, and F2, but was negatively correlated with the formation of the main flavor compounds, A9, A18, A20, C1, and G2. *L. sakei* was significantly positively correlated with the formation of the main flavor compounds, B4, B6, F1, and F2, but was negatively correlated with the formation of the main flavor compounds, A9, A15, A18, A20, and C1. *W. viridescens*, *W. paramesenteroides*, *L. fermentum*, *L. paralimentarius*, and *L. fallax* were significantly positively correlated with the formation of the main flavor compounds, B4, B6, F1 and F2, but were negatively correlated with the formation of the main flavor compounds, A9, A15, A18, A20, C1, and G2.

In 2017, FG samples were collected from Shanxi Xinghuacun Fenjiu Distillery Co., Ltd. (Fenyang, China) during light-flavored Baijiu production (Huang et al., 2020), and *L. brevis* was found to be positively correlated with A9 formation; however, *L. paralimentarius*, *Hanseniaspora u*var*um*, and *Bacillus licheniformis* were negatively correlated with A9 formation. In addition, *L. buchneri*, *L. odoratitofui*, and *L. parabuchneri* were positively correlated with A14 formation. *L. brevis* was positively correlated with A20 and B3 formation. Wu et al., 2021 found that esters present in Chinese liquor were produced by *A. oryzae*, *S. cerevisiae*, *S. pombe*, *S. fibuligera*, and *W. anomalus*, and that the main alcohol- and acid-producing microbes were *A. oryzae*, *A. hennebergii*, *S. cerevisiae*, *Z. bailii*, *P. kudriavzevii*, *S. pombe*, *L. homohiochii*, and *L. buchneri*. Furthermore, for the production of strong-flavored Baijiu, the most common hexanoate-producing *Clostridium* species identified included *C. kluyveri*, *C. lushun*, *Clostridium* spp. W1, and *C. celerecrescens*. Ethyl lactate-, ethyl acetate-, and ethyl butanoate-producing microbes in the strong-flavored Baijiu ecosystem were identified to be *Corynebacterium xerosis*, *Staphylococcus auricularis*, *Bacillus subtilis*, *Bacillus megaterium*, *Bacillus cereus* group, and *Paenibacillus macerans* (Zou et al., 2018).

### 4.4 Effect of proso millet on ethanol production and the abundance of key microbial communities for ethanol synthesis

Ethanol concentration is a direct reflection of the Baijiu yield and is significantly affected by sorghum variety and the microbial communities present during fermentation. A comparison of sorghum fermentation and mixed fermentation showed no significant difference in ethanol concentration at the final fermentation stage (28 days); however, ethanol concentration at the fermentation stages on days 14 and 21 were significantly lower for sorghum fermentation than for mixed fermentation (Table 2). The RDA analysis results showed that the presence of fungal species of the genera *Olipidium* was significantly positively correlated with ethanol production; however, the presence of fungi from the *Psathyrella* and *Schizothecium* genera was significantly negatively correlated with ethanol production (Supplementary Table S2). In total, 12 bacterial genera were significantly negatively correlated with ethanol production, with the negative correlation coefficient for the genus, *Brachybacterium*, being higher than that for other genera (−0.582; *p* < 0.01). In addition, the relative abundance of the bacteria of the genus, *Olipidium*, in mixed fermentation samples was lower than that in sorghum samples; however, the relative abundance of microbes that inhibit ethanol synthesis was higher in mixed fermentation samples than in sorghum samples (Figures 2, 3). Therefore, we speculated that proso millet could improve the flavor of light-flavored *Baijiu* but could not improve its yield. Furthermore, *L. buchneri*, *P. inopinatus*, *L. malefermentans*, *L. hokkaidonensis*, *P. pentosaceus*, *B. vulgatus*, *A. ruber*, and *O. truncatum* played important roles in ethanol production in proso millet Baijiu. Conversely, during the production of light-flavored *Xiaoqu* Baijiu, ethanol production was significantly positively correlated with the presence of the microbe, *Saccharomyces cerevisiae*, and negatively correlated with the presence of *A. pasteurianus* (Xie et al., 2021). *L. ramosa*, *Saccharomycopsis fibuligera*, *B. licheniformis*, *S. cerevisiae*, and *Pichia kudriavzevii* play significant roles in starch degradation and ethanol production (Huang et al., 2020). Furthermore, *S. cerevisiae*, *Z. bailii*, and *S. pombe* were found to play important roles in ethanol production in *Daqu* Chinese Baijiu (Wu et al., 2021); in addition, *S. cerevisiae* was the main ethanol-producing microbe identified during zaopei fermentation (Zou et al., 2018).

While the effects of proso millet on dynamic changes in microbial communities and flavor during different stages of light-flavored Baijiu fermentation using the light-flavored Baijiu technology of Xinghua Village, Shanxi, have been studied and some key microorganisms closely related to flavor changes identified, several important aspects still require further research. These include determining the optimal quantity of proso millet to be added, selecting superior proso millet varieties, investigating the interaction between proso millet quality and Baijiu flavor, and determining whether the addition of key flavor microorganisms can further enhance Baijiu quality. In addition, differences in flavor analysis results determined using GC × GC-TOF-MS and GC-IMS need to be further investigated.

## 5 Conclusion

In this study, we evaluated the changes in microbial communities and volatile flavor compounds in proso millet Baijiu, a new Baijiu product of Shanxi light-flavored Baijiu, as well as the relationship between them. Proso millet significantly changed the main flavor compound composition of light-flavored Baijiu from ethyl acetate, ethyl hexanoate, ethyl hexanoate dimer, ethyl butanoate, ethyl lactate, and butyl acetate to oct-2-ene, 2-butanol, propyl propanoate, 2-pentenal, and 4-methylpentanal. In addition, proso millet significantly changed the microbial communities in light-flavored Baijiu during fermentation, particularly during the early stages of fermentation (0–14 days). Furthermore, the formation of special flavor compounds in proso millet Baijiu was significantly correlated with the presence of fungi from the *Rhizopus*, *Papiliotrema*, *Wickerhamomyces*, *Aspergillus*, and *Thermoascus* genera but negatively correlated with the presence of bacteria from the *Weissella*, *Acinetobacter*, *Psychrobacter*, *Pseudarthrobacter*, *Bacteroides*, and *Saccharopolyspora* genera. The low alcohol production rate observed in Fenjiu may be due to the presence of fungi from *Psathyrella* genus and bacteria from the *Staphylococcus*, *Kroppenstedtia*, *Brevibacterium*, and *Acetobacter* genera, which exhibit a significantly high abundance during fermentation. Proso millet significantly changed the flavor of light-flavored Baijiu by inducing the formation of special microbial communities but did not increase alcohol concentration. This study lays a foundation for future research on improving the flavor of light-flavored Baijiu by modifying fermentation materials.

## Data availability statement

The original contributions presented in the study are publicly available. This data can be found at: https://www.ncbi.nlm.nih.gov/, PRJNA1014983, PRJNA1015004, PRJNA1013705, PRJNA1014132.

## Author contributions

JZ: Conceptualization, Data curation, Formal analysis, Funding acquisition, Methodology, Project administration, Writing – original draft, Writing – review & editing. ZG: Conceptualization, Data curation, Investigation, Methodology, Project administration, Supervision, Writing – review & editing.
