# Supplementary material for: Dynamic changes in microbial communities and flavor during different fermentation stages of proso millet Baijiu, a new product from Shanxi light-flavored Baijiu
Source: Front Microbiol. 2024 Jan 22;15:1333466. doi: 10.3389/fmicb.2024.1333466 (PMC10839113; doi:10.3389/fmicb.2024.1333466)
Supplement: Supplementary file 2 [file Data_Sheet_1.docx]

Supplementary Material

# Supplementary Figures and Tables

## Supplementary Figures

**
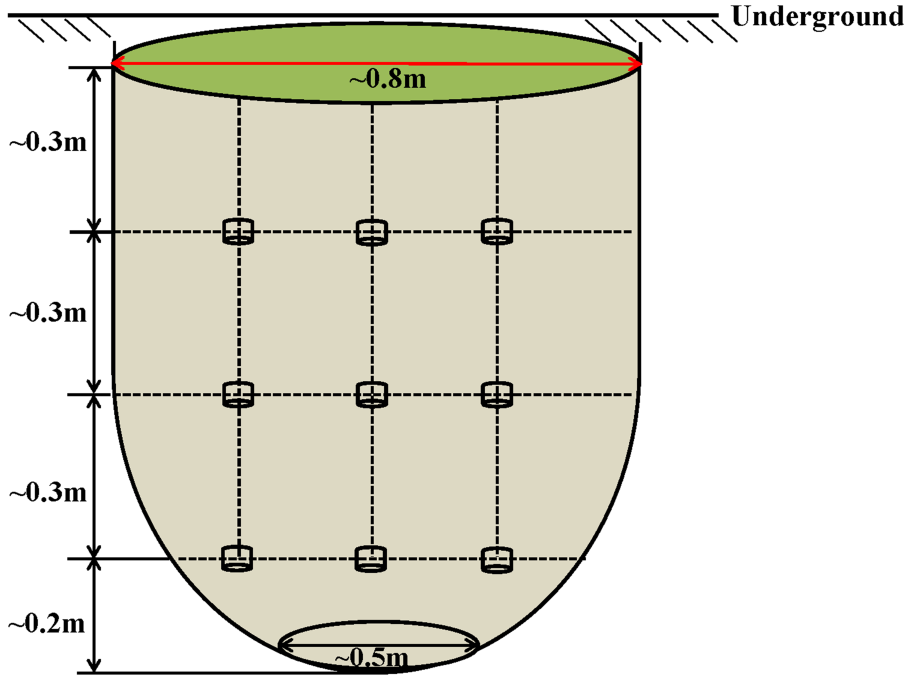
**

**Supplementary Figure 1.** Sampling design. Every fermented grain (FG) sample () was collected from nine different positions (100 g FG at each position) and mixed well as one sample.

## Supplementary Tables

**Supplementary Table 1.** The information of twenty unidentified compounds

| Compound | CAS# | Formula | RI | Rt [sec] | Dt [a.u.] | [+] MA-1 | [+] MA-2 | [+] MA-3 | [+] MB-1 | [+] MB-2 | [+] MB-3 | [+] MC-1 | [+] MC-2 | [+] MC-3 | [+] MD-1 | [+] MD-2 | [+] MD-3 | [+] GA-1 | [+] GA-2 | [+] GA-3 | [+] GB-1 | [+] GB-2 | [+] GB-3 | [+] GC-1 | [+] GC-2 | [+] GC-3 | [+] GD-1 | [+] GD-2 | [+] GD-3 |
| --- | --- | --- | --- | --- | --- | --- | --- | --- | --- | --- | --- | --- | --- | --- | --- | --- | --- | --- | --- | --- | --- | --- | --- | --- | --- | --- | --- | --- | --- |
| 1 | unidentified | * | 1185.5 | 626.856 | 0.93698 | 41.67808 | 40.21168 | 48.9942 | 34.55558 | 34.43179 | 33.26057 | 37.49789 | 36.91388 | 40.06885 | 33.84777 | 37.93274 | 49.94958 | 48.49905 | 62.17274 | 52.47928 | 49.25447 | 48.58475 | 40.30056 | 68.85723 | 88.89483 | 79.79491 | 98.78191 | 126.2912 | 90.85955 |
| 2 | unidentified | * | 1215.6 | 672.461 | 0.96571 | 99.43575 | 104.2444 | 98.50577 | 98.38833 | 106.1647 | 104.5047 | 93.65586 | 100.4038 | 98.48673 | 105.241 | 96.6331 | 117.7721 | 104.1301 | 104.1397 | 111.2844 | 124.8216 | 108.7706 | 122.9743 | 110.2528 | 116.877 | 107.1613 | 98.95966 | 110.2401 | 96.58549 |
| 3 | unidentified | * | 856.8 | 263.419 | 1.06483 | 554.0541 | 554.9999 | 538.7585 | 571.1208 | 547.3632 | 548.8677 | 531.3789 | 509.4908 | 518.3908 | 496.5472 | 589.0635 | 494.538 | 567.1279 | 538.0126 | 523.0947 | 604.8638 | 560.3005 | 564.6839 | 649.6969 | 647.6148 | 609.5518 | 710.6127 | 711.822 | 763.1745 |
| 4 | unidentified | * | 853 | 260.861 | 1.25271 | 133.1407 | 142.4692 | 146.5636 | 97.66782 | 93.932 | 93.09406 | 101.3497 | 102.978 | 113.0206 | 183.03 | 129.0367 | 185.4549 | 97.16316 | 109.0562 | 108.4055 | 167.9724 | 154.4892 | 166.6996 | 198.151 | 204.7435 | 204.7149 | 221.0294 | 215.3415 | 216.8333 |
| 5 | unidentified | * | 838.5 | 251.142 | 1.14093 | 1266.943 | 1246.127 | 1238.17 | 227.3425 | 304.5728 | 286.1508 | 343.1308 | 336.6177 | 313.879 | 339.2585 | 146.0399 | 317.9196 | 1357.355 | 1460.634 | 1535.906 | 192.6822 | 309.1466 | 265.7005 | 276.1939 | 276.5716 | 307.5278 | 332.063 | 332.647 | 310.7748 |
| 6 | unidentified | * | 803.8 | 227.96 | 1.13332 | 903.1171 | 840.8968 | 886.006 | 115.9438 | 129.2589 | 129.3541 | 176.3137 | 175.5266 | 183.8647 | 134.0612 | 178.0563 | 125.9769 | 1033.874 | 1081.415 | 1087.753 | 68.56204 | 98.9787 | 104.2571 | 107.9612 | 101.2735 | 108.7198 | 105.2474 | 111.059 | 102.2035 |
| 7 | unidentified | * | 855.2 | 262.342 | 1.22707 | 371.7319 | 395.334 | 367.8057 | 605.962 | 589.0985 | 586.6323 | 542.7228 | 516.4165 | 549.2518 | 625.3394 | 528.9698 | 625.3013 | 409.3727 | 417.3966 | 411.6325 | 680.8499 | 708.8607 | 696.8057 | 943.7161 | 936.9427 | 933.7719 | 1213.524 | 1285.431 | 1306.018 |
| 8 | unidentified | * | 871 | 272.949 | 1.05415 | 376.1882 | 356.5061 | 319.313 | 74.94183 | 64.05176 | 62.63932 | 63.48996 | 50.26698 | 58.3163 | 63.62962 | 84.86065 | 69.80943 | 68.4827 | 71.18378 | 68.01294 | 59.59861 | 68.9715 | 56.57059 | 67.56223 | 77.83971 | 63.63279 | 125.8912 | 108.1929 | 111.6526 |
| 9 | unidentified | * | 925.3 | 309.285 | 1.37269 | 719.8523 | 748.6216 | 756.7884 | 556.5552 | 573.6854 | 560.5736 | 760.4258 | 750.4784 | 783.6121 | 732.45 | 751.4306 | 747.0917 | 896.9183 | 880.9656 | 918.8793 | 623.4921 | 660.2029 | 623.3398 | 727.1684 | 736.481 | 691.6543 | 716.4465 | 729.622 | 750.1388 |
| 10 | unidentified | * | 966.1 | 336.547 | 1.3943 | 566.9597 | 569.1148 | 526.7988 | 1040.99 | 1024.051 | 1020.975 | 1207.042 | 1158.074 | 1183.475 | 1190.807 | 1220.138 | 1231.46 | 343.9688 | 388.3448 | 314.4123 | 1169.297 | 1124.042 | 1163.279 | 1173.658 | 1197.365 | 1162.241 | 1300.663 | 1182.653 | 1218.374 |
| 11 | unidentified | * | 989.7 | 352.391 | 1.26968 | 341.2391 | 354.6715 | 351.2214 | 375.417 | 375.5598 | 380.9683 | 359.2898 | 371.6748 | 371.3479 | 416.146 | 384.7137 | 420.8118 | 356.941 | 383.771 | 370.8464 | 451.8062 | 444.0044 | 461.4425 | 466.1654 | 480.6358 | 495.103 | 427.9565 | 439.967 | 357.7186 |
| 12 | unidentified | * | 989.7 | 352.391 | 1.40478 | 725.6132 | 719.2238 | 678.5869 | 1133.281 | 1117.38 | 1111.197 | 1157.448 | 1104.607 | 1133.932 | 1265.8 | 1198.219 | 1265.575 | 370.0687 | 405.0147 | 345.889 | 1280.515 | 1290.77 | 1325.249 | 1266.847 | 1300.889 | 1292.585 | 1309.151 | 1294.1 | 1264.34 |
| 13 | unidentified | * | 1042.9 | 408.497 | 1.26644 | 4604.709 | 4597.368 | 4621.319 | 4174.883 | 4193.524 | 4141.994 | 3808.871 | 3799.228 | 3787.532 | 4103.734 | 4141.264 | 3994.488 | 5255.787 | 5262.42 | 5211.931 | 4171.261 | 4241.944 | 4224.188 | 3966.87 | 4073.098 | 4093.349 | 3682.964 | 3774.541 | 3405.456 |
| 14 | unidentified | * | 1043.5 | 409.177 | 1.38316 | 579.1765 | 597.7794 | 600.0584 | 676.4602 | 680.5134 | 688.9183 | 655.0895 | 683.7256 | 660.679 | 761.4796 | 807.5187 | 805.3922 | 640.7525 | 683.0146 | 621.0926 | 754.1508 | 738.7441 | 742.6323 | 807.7061 | 841.2142 | 871.5642 | 783.7644 | 736.6492 | 619.1088 |
| 15 | unidentified | * | 1054.3 | 421.419 | 1.20051 | 212.2659 | 184.3884 | 216.8619 | 204.169 | 203.7119 | 207.124 | 198.0971 | 220.6517 | 195.7356 | 187.8608 | 246.2025 | 228.9168 | 515.4072 | 603.6228 | 715.5928 | 241.2923 | 229.5707 | 247.4087 | 256.7816 | 268.4969 | 286.9094 | 268.9984 | 230.4753 | 171.1115 |
| 16 | unidentified | * | 1069.7 | 438.761 | 1.274 | 68.71758 | 62.53458 | 77.74449 | 52.92047 | 55.22481 | 45.73131 | 47.03901 | 49.14973 | 52.43167 | 71.40279 | 72.07569 | 78.51578 | 825.6393 | 488.7803 | 720.8236 | 38.02478 | 37.80578 | 36.44412 | 42.72868 | 40.32277 | 45.96618 | 48.15626 | 45.8297 | 37.85339 |
| 17 | unidentified | * | 1291.8 | 781.799 | 1.23294 | 390.4047 | 492.1353 | 479.0393 | 216.7825 | 116.6612 | 96.15064 | 116.1597 | 104.9966 | 82.73088 | 78.37612 | 72.01538 | 74.76726 | 1232.133 | 1526.413 | 1442.999 | 717.9923 | 329.5523 | 166.36 | 133.2899 | 89.60581 | 94.50967 | 93.21468 | 85.50497 | 70.47598 |
| 18 | unidentified | * | 967.5 | 337.52 | 1.2645 | 454.5739 | 452.5425 | 452.1394 | 645.5357 | 647.1037 | 637.0167 | 718.8969 | 715.6055 | 724.1372 | 713.2662 | 707.4577 | 742.902 | 464.2229 | 511.4905 | 454.8342 | 704.8804 | 724.1086 | 706.0739 | 765.1456 | 781.3966 | 858.5888 | 720.0269 | 736.7 | 629.1927 |
| 19 | unidentified | * | 1141.1 | 547.701 | 1.277 | 62.93133 | 59.07489 | 70.20619 | 72.18678 | 67.12104 | 73.17707 | 85.18757 | 89.90099 | 88.19019 | 79.91552 | 80.26466 | 90.43105 | 72.32327 | 71.76781 | 80.14088 | 114.3029 | 106.5233 | 101.6449 | 143.7292 | 132.4678 | 141.0186 | 128.4432 | 145.3289 | 129.0938 |
| 20 | unidentified | * | 1140.8 | 547.153 | 1.69889 | 65.50546 | 76.3511 | 78.29677 | 114.7568 | 121.638 | 115.4265 | 129.8683 | 142.9643 | 131.5283 | 138.0255 | 150.8962 | 177.085 | 74.57682 | 91.51022 | 84.31471 | 215.5478 | 187.4419 | 172.451 | 237.7565 | 237.2645 | 257.4608 | 226.9489 | 250.1288 | 191.8728 |

**Supplementary Table 2.** The correlation between flavor compounds and fungal (F) or bacterial (B) abundance at the genus (I) or species (II) level
